# Synaptic plasticity-dependent competition rule influences memory formation

**DOI:** 10.1038/s41467-021-24269-4

**Published:** 2021-06-24

**Authors:** Yire Jeong, Hye-Yeon Cho, Mujun Kim, Jung-Pyo Oh, Min Soo Kang, Miran Yoo, Han-Sol Lee, Jin-Hee Han

**Affiliations:** 1 Department of Biological Sciences, Korea Advanced Institute of Science and Technology (KAIST), 291 Daehak-ro, Yuseong-gu, Daejeon, Korea; 2 KAIST Institute for the BioCentury (KIB), Korea Advanced Institute of Science and Technology (KAIST), 291 Daehak-ro, Yuseong-gu, Daejeon, Korea

**Keywords:** Fear conditioning, Long-term memory

## Abstract

Memory is supported by a specific collection of neurons distributed in broad brain areas, an engram. Despite recent advances in identifying an engram, how the engram is created during memory formation remains elusive. To explore the relation between a specific pattern of input activity and memory allocation, here we target a sparse subset of neurons in the auditory cortex and thalamus. The synaptic inputs from these neurons to the lateral amygdala (LA) are not potentiated by fear conditioning. Using an optogenetic priming stimulus, we manipulate these synapses to be potentiated by the learning. In this condition, fear memory is preferentially encoded in the manipulated cell ensembles. This change, however, is abolished with optical long-term depression (LTD) delivered shortly after training. Conversely, delivering optical long-term potentiation (LTP) alone shortly after fear conditioning is sufficient to induce the preferential memory encoding. These results suggest a synaptic plasticity-dependent competition rule underlying memory formation.

## Abstract (web summary)

Cellular activity level at the time of learning is thought to be a critical factor to determine which neurons are recruited to encode memory. Here, the authors show that competitive synaptic plasticity mechanisms influence which neurons will encode a fear memory.

## Introduction

Memory is thought to be supported by a sparse collection of neurons broadly distributed across multiple brain areas that forms a unique memory trace, referred to as engram^1^. Many studies over the past decade have identified ensembles of neurons and synapses where engram is thought to be localized. These cells and synapses are the brain sites where learning-dependent physical changes occur and are crucial for the later retrieval of memory^2–7^. Despite these recent advances in identifying an engram, how the engram is formed in the first place during memory formation is not well understood. Evidence from a series of previous studies suggests that neurons that participate in encoding memories are not hard-wired but rather selected by specific mechanisms. It has been previously shown that neurons with higher levels of cyclic AMP-response element binding protein (CREB) or cellular excitability than their neighboring cells at the time of learning are preferentially recruited to support memory^8–11^. So, a cellular activity level at the time of learning is thought to be a critical factor to determine which neurons are recruited to encode memory. Strengthening of synaptic connections between neurons that are active during an event is thought to be involved in the formation of engram^3,12,13^. Activity-dependent synaptic plasticity such as LTP or LTD mediates enduring changes in synaptic strength induced by learning, which are thought to be a mechanism for memory encoding and storage^14,15^. Thus, it is generally agreed that synaptic plasticity is critical for forming engram. However, the relation between synaptic plasticity and formation of memory encoding-neuronal ensembles is unclear. If synaptic plasticity is a mechanism for encoding of memory, we hypothesized that at which synapses the plasticity-related activities occur during memory formation may influence which cells and synapses are recruited to encode memory. Investigation of this idea, however, has been challenging due to the limitation of currently available engram labeling methods, which allow for targeting engram only after memory encoding or consolidation because labeling is mainly based on the gene expression driven by the activation of promoters of immediate early genes such as c-Fos and Arc. To overcome this limitation, here we attempted to target the synapses that are normally not potentiated by learning and applied optogenetic tools to these synapses to manipulate them to be potentiated by learning. By allowing for the manipulation of these synapses with optogenetic tools over the course of memory formation even right after learning, this approach enabled us to explore the role of plasticity-related synaptic activities for the construction of engram during memory formation. In this study, we show changes in cell ensembles that participate in encoding a fear memory in a competitive manner by a specific pattern of optogenetic synaptic stimulations related to synaptic plasticity delivered right before or after learning.

## Results

### LTP induction at the optogenetically primed auditory inputs to the LA by FC

**Fig. 1:**
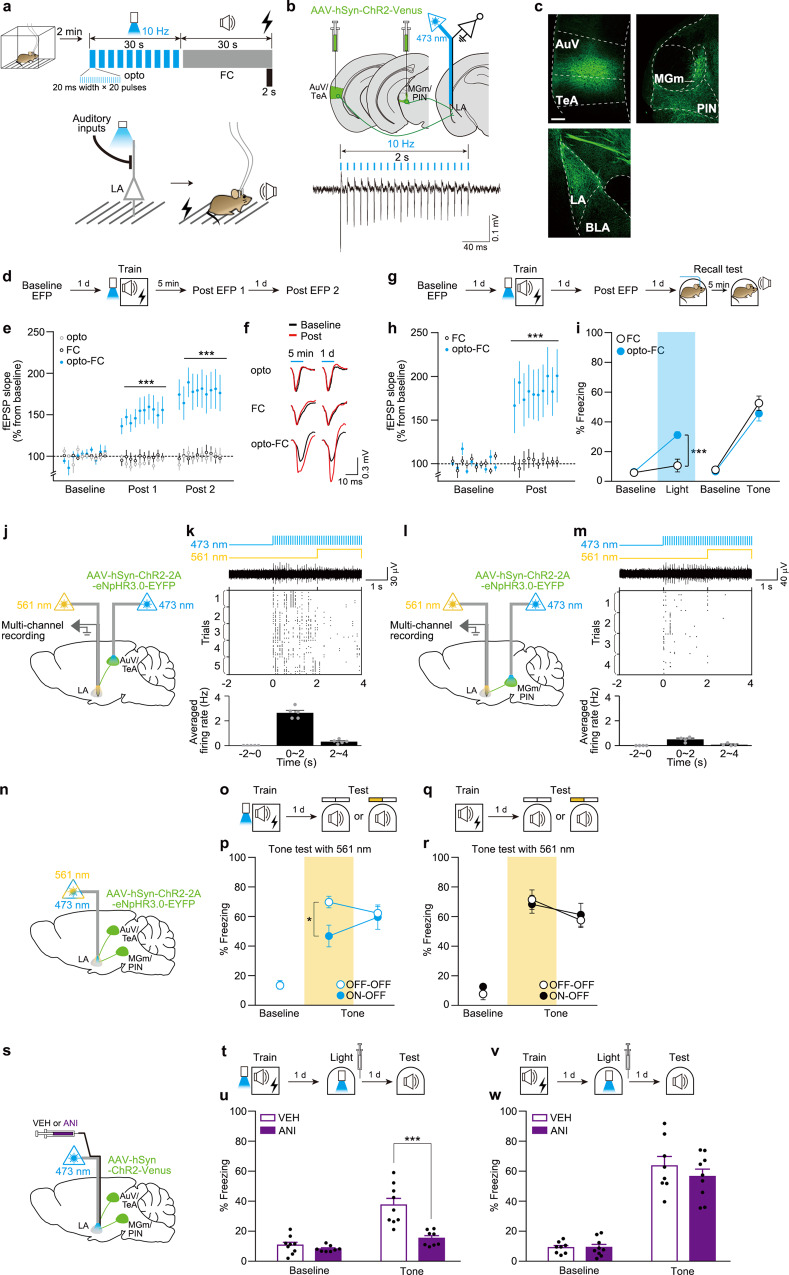
LTP induction and fear memory encoding at the auditory-to-LA synapses primed with an optical priming stimulus delivered immediately before fear conditioning. **a** Schematic of experimental procedure for optogenetic priming. A 10-Hz light pulse was delivered to the auditory axonal projections expressing ChR2 immediately before FC. **b** Top, schematic of AAV injection into the auditory regions and optrode implantation above the LA for light delivery and EFP recordings. Bottom, EFP trace evoked by 10-Hz opto-stimulation (473 nm, 20 ms) for 2-s duration. **c** Representative confocal microscopic images showing ChR2-Venus expression. Scale bar, 200 μm. **d** Experimental procedure for unilateral in vivo EFP recordings in the anesthetized animals. **e** Average of in vivo fEPSP slope (normalized to baseline) 1 d before (baseline), 5 min (post 1) and 1 d (post 2) after training in each group (opto, *n* = 8; FC, *n* = 9; opto-FC, *n* = 8; ***P < 0.0001, Tukey’s post hoc test). **f** Representative EFP traces. **g** Experimental procedure for EFP recordings and behavioral tests. **h** Average of in vivo fEPSP slope 1 d before (baseline) and 1 d after (post) training (*n* = 7 mice per group; ***P < 0.0001, Sidak’s post hoc test). **i** Freezing levels to input photoactivation (blue shaded) and tone. There was no significant difference in freezing to tone between groups (*n* = 7 mice per group; ***P = 0.0005, Sidak’s post hoc test). **j**, **l** Schematic of in vivo multiunit recording combined with optogenetic excitation or inhibition in mice with unilateral viral injection into the AuV/TeA (**j**) or MGm/PIN (**l**) and optic fiber implantation. **k**, **m** Top, light stimulation procedure. Middle, representative multiunit activity trace and raster plot of activities over threshold. Bottom, averaged firing rate during each 2-s time bin (*n* = 5 (**k**) or 4 (**m**) independent trials from different brain regions in *n* = 2 mice). **n** Schematic of optogenetic activation and inhibition of AAV-expressing axonal inputs in the bilateral LA. **o**–**r** Behavioral procedure (**o**, **q**). The freezing level was measured during tone with (ON, yellow-shaded) or without (OFF) 561 nm light in opto-FC (**p**) and FC control mice (**r**) (*n* = 8 mice per group; *P = 0.0323, Tukey’s post hoc test). **s** Schematic of opto-stimulation and drug delivery in the bilateral LA. **t**–**w** Behavioral procedures (**t**, **v**). Amnesic effect of anisomycin was observed in opto-FC (VEH, *n* = 9; ANI, *n* = 8; ***P < 0.0001, Sidak’s post hoc test) (**u**) but not in FC condition (VEH, *n* = 8; ANI, *n* = 9) (**w**). VEH vehicle injection, ANI anisomycin injection. Data are mean ± s.e.m. Two-way repeated-measures ANOVA (**e**, **h**, **i**, **p**, **r**, **u**, **w**).

Pavlovian fear conditioning has been greatly useful and thus widely used as a model for simple associative learning^16^. During auditory cued fear conditioning (hereafter FC), an emotionally neutral tone (conditioned stimulus, CS) is paired with an aversive footshock (unconditioned stimulus, US). As a result of the association, the tone acquires an ability to evoke fear-related behavioral responses such as freezing, an index of fear memory. It has been well established that associative plasticity, a form of LTP induced at the weak presynaptic inputs coactive with a strong depolarization of postsynaptic neurons, at auditory-to-LA synapses encodes fear memory^17,18^. We targeted these auditory synaptic connections for our investigations. In order to manipulate LTP induction by learning at these synapses, we adopted a priming paradigm, a form of metaplasticity. Neural activity at one point in time can change cells or synapses such that their ability to exhibit LTP or LTD after a later bout of activity such as learning is altered. This form of plasticity regulation has been termed metaplasticity^19^. We examined whether the LA synapses can be primed for LTP induction by using a pattern of optogenetic synaptic stimulation that precedes FC (Fig. 1a). Previously, we reported that a pattern of 10-Hz optogenetic stimulation of both thalamic and cortical inputs in the LA simultaneously can drive an associative fear memory formation as a CS when paired with a footshock^20^. In this case, the optical stimulation and footshock were paired six times. Although not published previously, we also observed that a single pairing does not drive fear memory formation. Based on this preliminary observation, we chose such a specific pattern of optogenetic stimulation protocol (see “Methods”) and tested whether it can act as a priming stimulus. We injected an adeno-associated virus (AAV) encoding channelrhodopsin-2 (ChR2) fused with Venus driven by the human synapsin promoter (AAV-hSyn-ChR2-Venus) into the secondary auditory and temporal association cortex (AuV/TeA) and medial geniculate nucleus (MGm/PIN) (Fig. 1b, c)^20^. An optrode was placed above the LA for light delivery and recording of light-evoked field potentials (EFPs). During training, mice were placed in the conditioning chamber and allowed for free exploration for 2 min. We then delivered the 10-Hz opto-stimulation (473 nm, 20-ms pulse width) as a potential priming stimulus to the ChR2-expressing axonal projections for 30 s immediately before FC and examined whether LTP is induced at these opto-stimulated inputs (Fig. 1a, d). LTP induction was determined by measuring field population excitatory postsynaptic potential (fEPSP) evoked by the light activation of ChR2-expressing auditory inputs to the LA (Fig. 1d–f). All EFP recordings were conducted in anesthetized animals in this study. We found that LTP was elicited 5 min and 1 d after training in mice received the opto-stimulation before FC (opto-FC, Fig. 1e, f). This LTP induction was not due to the opto-stimulation itself (opto, Fig. 1e, f). Importantly, without the priming stimulus, LTP was not detected at the ChR2-expressing auditory inputs after FC in our training condition (non-discriminative, simple tone for the CS, and 1 trial) (FC, Fig. 1e, f). To further validate the LTP induction by priming, we tested whether the 10-Hz stimulation used as the priming stimulus induces LTP or long-term memory (LTM) formation when paired with footshock (opto-trace-shock condition, Supplementary Fig. 1a, b). We found no LTP induction and LTM formation in this condition (Supplementary Fig. 1c–e), thus excluding this possibility. Consistent with no LTM formation, we observed no significant induction of the immediate early genes (IEGs) c-Fos and Arc in the LA in this opto-trace-shock condition. The number of c-Fos and Arc-positive signals in the LA of the opto-trace-shock group was comparable to that of a homecage control (HC) but significantly less than that of a fear conditioning control (FC) (Supplementary Fig. 1f–j). Collectively, these results establish that we targeted the auditory-to-LA synapses that were not potentiated by FC and manipulated these synapses to be potentiated by FC in our condition.

The AAV 2/1 serotype virus used in this study can be transsynaptically transported in either anterograde or retrograde direction^21,22^. So, it was possible that ChR2 was expressed in the LA neurons, although the AAV virus was injected into the presynaptic auditory regions. From the histological analysis conducted after experiments to check the virus expressions, we have never seen any ChR2-expressing cells in the LA (Fig. 1c). To further confirm this and systematically characterize the gene expression patterns by the AAV virus in our condition, we performed additional imaging analysis. To clearly identify the virus-expressing neurons, we injected AAV-hSyn-EGFP virus unilaterally into the AuV/TeA and MGm/PIN and quantified the number of EGFP-expressing neurons in the virus injected sites (auditory regions) and LA. Consistent with our observation with ChR2, we again did not find any EGFP-positive cells in the LA throughout all brain sections we examined (Supplementary Fig. 2), indicating no detectable transneuronal transports by the AAV 2/1 virus in our virus injection condition. These results are consistent with the previous report that the efficiency of the transneuronal spread by AAV1 is very low, leading to extremely weak transneuronal expression in the downstream neurons without an amplification step^22^. Notably, cell-counting analysis in the AuV/TeA and MGm/PIN showed that EGFP was expressed in a sparse population of neurons (<15%) in these regions (Supplementary Fig. 2).

### Memory encoding at the primed auditory inputs

Next, we explored the encoding of fear memory at the manipulated inputs. We first confirmed that fear memory formation was normal in this priming condition. We performed both EFP recordings and behavioral tests on the same animal (Fig. 1g–i). One day after post learning EFP recordings, animals were tested with a photostimulation of the inputs and subsequently with a conditioned tone. As before, LTP was induced in the opto-FC condition but not in the control FC (Fig. 1h). Animals in the opto-FC group displayed significant freezing to the photostimulation of the inputs compared to baseline level but not in the control group. Animals from both groups displayed similar level of freezing to the tone CS (Fig. 1i). To determine memory encoding at the targeted auditory synapses, we examined whether fear memory retrieval depends on these synapses. To this end, we optogenetically silenced these synapses during memory retrieval. We employed a bidirectional optogenetic manipulation by using the AAV encoding both ChR2 and halorhodopsin (eNpHR3.0) under the human synapsin promoter (AAV-hSyn-ChR2-2A-eNpHR3.0-EYFP)^23,24^. We verified this approach using in vivo multiunit recording in the LA of anesthetized mice combined with optogenetic stimulations in two different locations (Fig. 1j, l). The 473-nm blue light illumination (activating ChR2) to the soma of neurons infected with the AAV in the AuV/TeA (Fig. 1j) or MGm/PIN (Fig. 1l) induced an increase in firing rate in the LA neurons. This increase, however, was suppressed by simultaneously delivered 561-nm yellow light (activating eNpHR3.0) to their axonal projections in the LA (Fig. 1k, m). For the behavioral tests, we injected the AAV-hSyn-ChR2-2A-eNpHR3.0-EYFP virus into the bilateral auditory regions, and chronically implanted an optic fiber above the LA for light delivery (Fig. 1n). We trained these mice with opto-FC or FC protocol. Next day, mice were tested with tone under two different experimental conditions (Fig. 1o, q). In ON-OFF condition, yellow light was delivered for optical silencing during the first 1-min of tone presentation followed by another 1-min of tone without the light. In OFF-OFF condition, the only tone was presented without light during entire 2-min of the test session. Compared with OFF-OFF control group, silencing the manipulated inputs caused significantly reduced freezing to the tone in ON-OFF group (Fig. 1o, p), indicating a disruption of memory retrieval by silencing. This effect was specific to silencing such that there was no significant group difference in freezing during the light OFF period. It was also specific to the priming condition. Without the priming procedure, the input silencing alone did not affect memory retrieval as shown in FC control condition (Fig. 1q, r). Moreover, when we used different opto-stimulation protocols during the priming period by which LTP was not induced at the opto-stimulated synapses, the silencing did not affect memory retrieval (Supplementary Fig. 3).

We next tested whether artificially stimulating these synapses may activate auditory fear memory retrieval. Upon retrieval, memory is known to enter a labile state vulnerable to amnesic agents such as anisomycin, a protein synthesis blocker^25,26^. Using naive animals, we confirmed such amnesic effect of anisomycin on fear memory in our condition. Immediately after fear memory retrieval test by the conditioned tone, animals were administered with either anisomycin or vehicle as a control (Supplementary Fig. 4a). Animals in the anisomycin group, but not in the control group, displayed a significantly reduced freezing to the tone during Test 2 (after anisomycin) compared with Test 1 (before anisomycin) (Supplementary Fig. 4b). Based on this reconsolidation concept, we tested whether auditory fear memory becomes vulnerable to anisomycin by artificially stimulating the targeted auditory inputs. We injected the AAV-hSyn-ChR2-Venus in the bilateral auditory regions. For the delivery of light and drug to the same sites in the LA, a custom-made fluid-optic cannula was placed above the bilateral LA (Fig. 1s). One day after opto-FC training, mice received either anisomycin or vehicle immediately after photostimulation of the inputs. The next day, these mice were tested with tone (Fig. 1t). Animals in the anisomycin group displayed significantly less freezing to tone compared with the control group (Fig. 1u). This amnesic effect was specific. When anisomycin was treated without the photostimulation, memory was not impaired (Supplementary Fig. 4c–e). Moreover, in no priming control group (FC alone), anisomycin did not produce the amnesic effect even with the photostimulation (Fig. 1v, w).

### LTD at the primed inputs 24 h after training disrupts fear memory retrieval

**Fig. 2:**
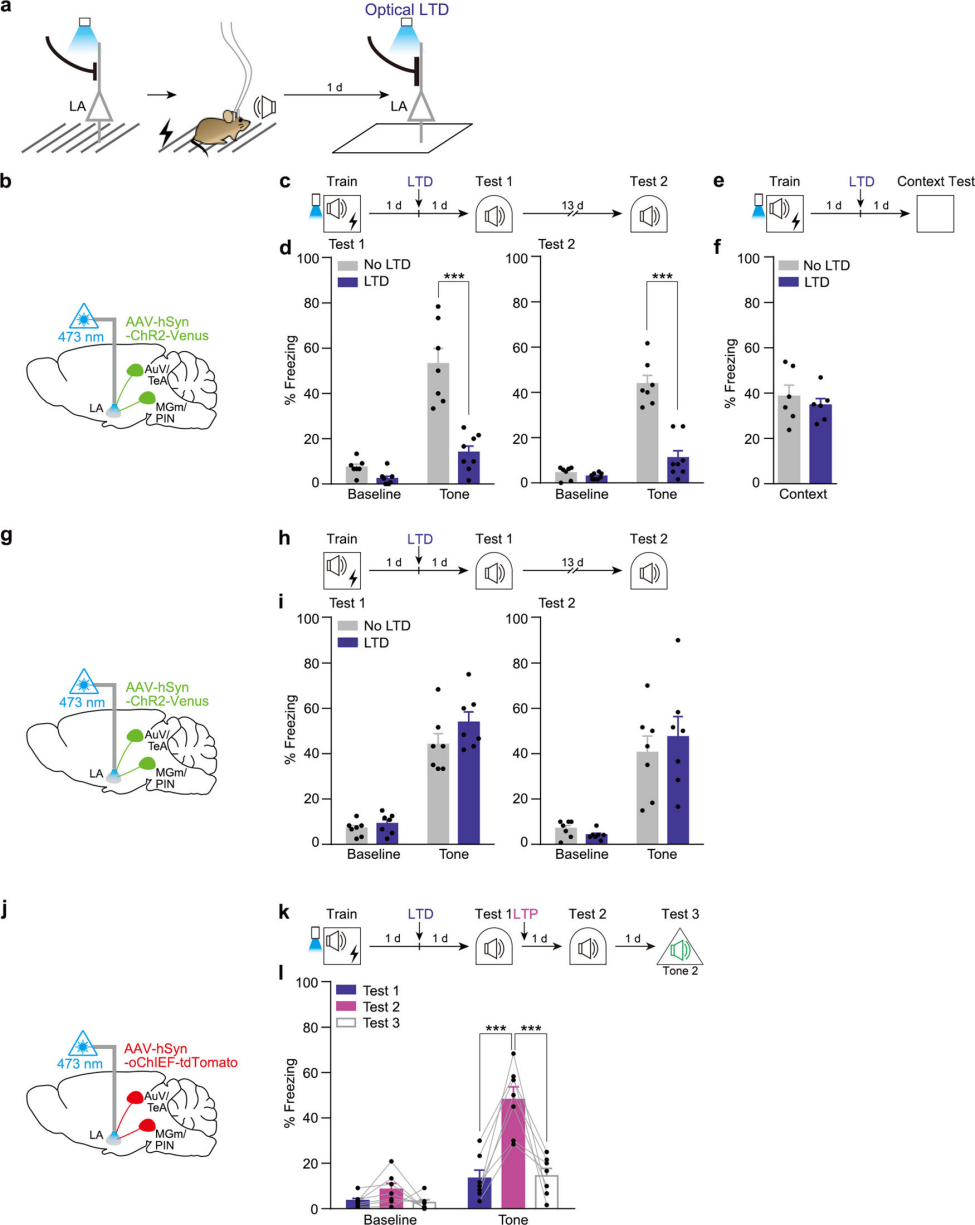
Optical LTD delivered to the primed synapses 24 h after training disrupts fear memory retrieval. **a** Schematic of behavior paradigm. **b**, **g**, **j** AAV-hSyn-ChR2-Venus (**b**, **g**) or AAV-hSyn-oChIEF-tdTomato (**j**) was injected into the bilateral AuV/TeA and MGm/PIN with optic ferrule implantation above the LA. **c** Behavioral procedures. Optical LTD was delivered 24 h after training. **d** Freezing levels to tone were significantly reduced in LTD group (*n* = 8 mice) compared to the control group (*n* = 7 mice) during both Test 1 and Test 2 (***P < 0.0001, Sidak’s post hoc test). **e** Behavioral procedure for a contextual fear memory test. **f** Freezing levels to context. There was no significant group difference in freezing (*n* = 6 mice per group). **h** Behavioral procedures. LTD experiment in control FC condition. **i** There was no significant difference in freezing level to tone between groups (*n* = 7 mice per group). **k** Behavioral procedures. Optical LTP was delivered successively 1 d after optical LTD in opto-FC. Tone2 was presented as an unconditioned distinct tone in a distinct context. **l** Freezing level to tone was measured during Tests. Tone2 was presented as an unconditioned distinct tone in a distinct context (*n* = 7 mice; ***P < 0.0001, Sidak’s post hoc test). Data are mean ± s.e.m. Two-way repeated-measures ANOVA (**d**, **i**, **l**). Two-tailed unpaired *t* test (**f**).

We next examined the effect of LTD or LTP induction on fear memory formation. To this end, we used previously established optogenetic stimulation protocols for the induction of LTD or LTP^2,7^. Because oChIEF can follow 50–100 Hz and hence induce LTP by high-frequency stimulation protocol but ChR2 cannot^2,27^, we used the oChIEF for all LTP experiments. We confirmed the induction of LTD or LTP by each protocol in our hand by conducting in vivo EFP recordings in the LA of anesthetized mice injected with the AAV-hSyn-oChIEF-tdTomato in the unilateral auditory regions (Supplementary Fig. 5). We delivered the optical LTD to the primed synapses 24 h after training (Fig. 2a). Animals in the LTD group displayed dramatically reduced freezing to the conditioned tone compared with a control group up to 14 d after the LTD delivery (Fig. 2b–d). This effect was memory-specific as the same optical LTD did not affect contextual fear memory (Fig. 2e, f). Importantly, the optical LTD did not affect retrieval in control animals trained with FC (Fig. 2g–i). Similar to a previous report^2^, we found that memory impaired by optical LTD was reactivated by subsequently delivered optical LTP in our condition (Fig. 2j–l). To test the specificity of optical LTP effect on memory retrieval, the same mice were tested again but this time with a distinct tone (Tone2) that was not associated with shock (Supplementary Fig. 6). In response to the Tone2 during Test 3, mice displayed significantly lower freezing levels compared with the levels measured during Test 2 (Fig. 2l). As controls, the optical LTP alone or delivered immediately following a tone did not produce freezing to the tone (Supplementary Fig. 7). Taken together, these results demonstrate that fear memory was encoded at the primed auditory inputs for LTP induction.

### LTD shortly after training abolishes a preferential memory encoding in the manipulated neuronal ensembles

**Fig. 3:**
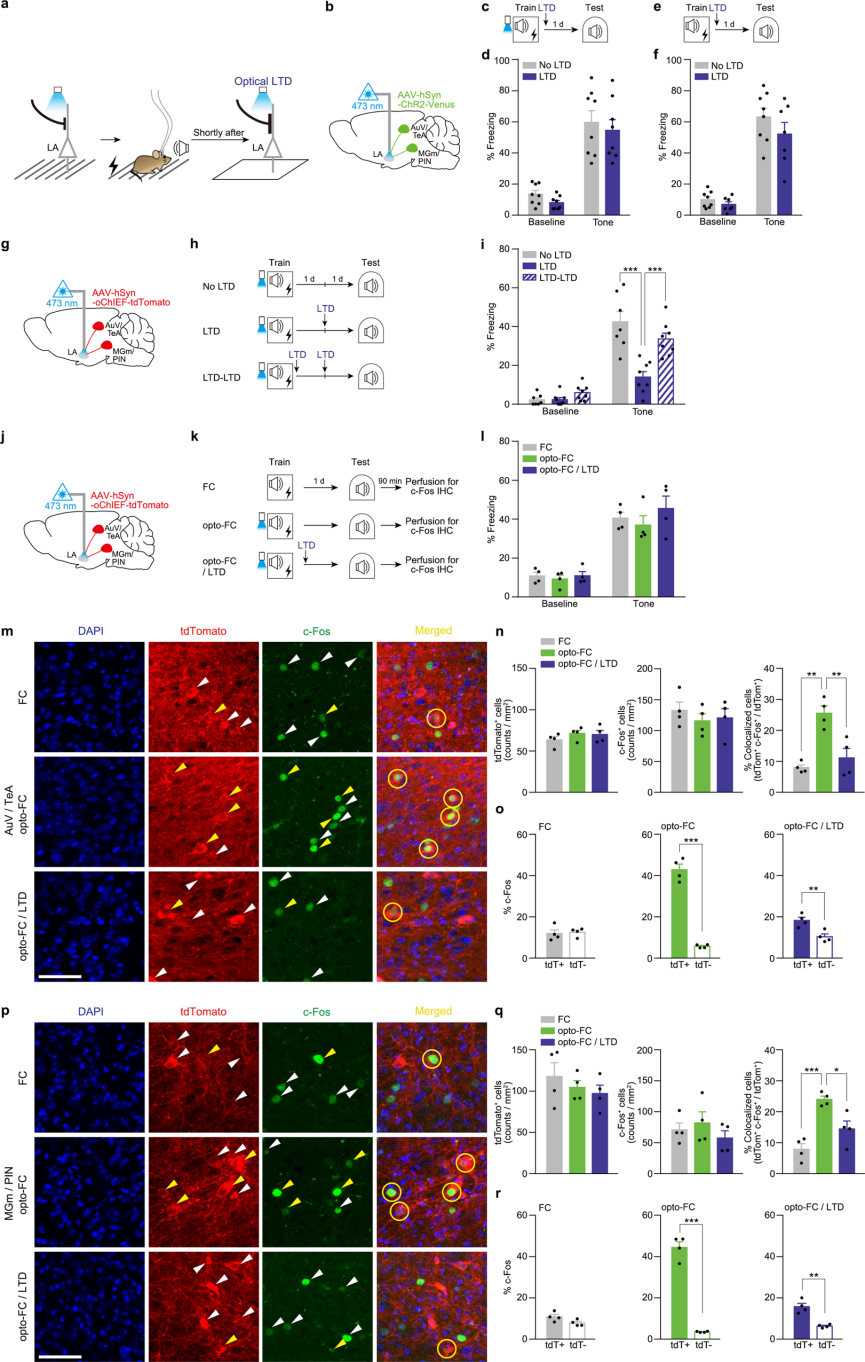
Fear memory is preferentially encoded in cell ensembles manipulated with priming, which is abolished with optical LTD delivered shortly after training. **a** Schematic of behavior paradigm. Optical LTD was delivered shortly (about 5 min) after opto-FC. **b**, **g**, **j** AAV-hSyn-ChR2-Venus (**b**) or AAV-hSyn-oChIEF-tdTomato (**g**, **j**) was injected into the bilateral AuV/TeA and MGm/PIN with optic ferrule implantation above the LA. **c**, **e** Behavioral procedures. Optical LTD was delivered shortly (about 5 min) after opto-FC (**c**) or FC (**e**) training. **d**, **f** Freezing levels to tone measured during Test in opto-FC (*n* = 8 mice per group) (**d**) or FC (No LTD, *n* = 8 mice; LTD, *n* = 7 mice) (**f**) condition. **h** Behavioral procedures. **i** Freezing levels to tone measured during Test (No LTD, *n* = 7 mice; LTD and LTD-LTD, *n* = 8 mice per group; ***P < 0.0001, Sidak’s post hoc test). **k** Behavioral procedures. Brains were taken 90 min after the test for immunostaining. **l** Freezing levels to tone measured during the test (*n* = 4 mice per group). **m**, **p** Representative confocal microscopic images in the AuV/TeA (**m**) and MGm/PIN (**p**). Similar results were obtained from four independent mice per group, and the results were quantified in (**n**), (**o**), (**q**), (**r**). White arrows indicate tdTomato^+^ or c-Fos^+^ cells. Yellow arrows and circles indicate colocalized cells. Scale bar, 50 μm. **n**, **o**, **q**, **r** Quantitative analysis for distribution pattern of tdTomato^+^ and c-Fos^+^ cells in the AuV/TeA (**n**, **o**) and MGm/PIN (**q**, **r**). **n**, **q** Cell counts of tdTomato^+^ (left), c-Fos^+^ (middle), and tdTomato^+^/c-Fos^+^ overlap (right; FC vs. opto-FC **P = 0.001, opto-FC vs. opto-FC / LTD **P = 0.0038 (**n**); FC vs. opto-FC ***P = 0.0006, opto-FC vs. opto-FC / LTD *P = 0.016 (**q**); Tukey’s post hoc test). **o**, **r** c-Fos^+^ cells were more likely to be colocalized to tdTomato^+^ cells than neighboring tdTomato^-^ cells in opto-FC (middle) and opto-FC/LTD (right), but evenly distributed in FC control group (left). (*n* = 4 mice per group; ***P < 0.0001, **P = 0.0086 (**o**), ***P < 0.0001, **P = 0.0013 (**r**); two-tailed unpaired *t* test). Data are mean ± s.e.m. Two-way repeated-measures ANOVA (**d**, **f**, **i**, **l**). One-way ANOVA (**n**, **q**). Two-tailed unpaired *t* test (**o**, **r**).

Because we aimed to explore the role of plasticity-related activities for the construction of engram during memory formation, we then delivered the optical LTD shortly (~5 min) after training (Fig. 3a). Interestingly, in this case, we found no significant effect on memory retrieval, indicating an intact fear memory formation in this condition (Fig. 3b–d). In control experiments, we also found normal fear memory formation (Supplementary Fig. 8 and Fig. 3e, f). In contrast, when the optical LTD was delivered at a slightly later time point (30 min) after training, fear memory retrieval was impaired as in the case of the optical LTD delivered 24 h after training (Supplementary Fig. 9). We hypothesized that these behavior results may be explained if encoding was not established yet shortly after training and the optical LTD affected the encoding process, leading to a change in the engram. To explore this possibility, we first examined whether these synapses exposed to the optical LTD shortly after training are still involved in encoding of fear memory. For this purpose, we delivered the optical LTD again to these synapses 24 h after training and determined its effect (Fig. 3g, h). As before, memory was impaired in control animals by the optical LTD delivered 24 h after training. However, the same LTD delivery did not affect memory in animals that were exposed to the optical LTD shortly after training (Fig. 3i), suggesting that fear memory encoding at the primed synapses was abolished due to the LTD delivered shortly after training. Because memory formation was normal, it was assumed that there might have been a change in cell populations that participate in encoding fear memory in this condition. To test this possibility, we performed an imaging analysis by detecting neuronal activation in the AuV/TeA and MGm/PIN during memory retrieval. Using c-Fos expression as a marker for recently activated neurons (c-Fos^+^), we determined the probability of activation of the labeled neurons (tdTomato^+^) during memory retrieval (Fig. 3j, k). Animals used for imaging analysis displayed no significant difference in freezing response to tone between groups during the test (Fig. 3l). Imaging analysis showed that, compared with a control group (FC), the overlap ratio between tdTomato^+^ and c-Fos^+^ cell population was significantly higher in the opto-FC group in both AuV/TeA and MGm/PIN (Fig. 3m, n, p, q). This increase, however, was reversed in mice that received the optical LTD shortly after training (Fig. 3n). Despite these changes in overlap, there was no significant difference in the overall size of tdTomato^+^ and c-Fos^+^ cell populations across all conditions (Fig. 3n, q). Furthermore, the proportion of c-Fos^+^ cells was higher in tdTomato^+^ cells than in their noninfected neighbors in opto-FC, while the distribution was even in FC control (Fig. 3o, r). This bias in c-Fos^+^ distribution became smaller in opto-FC/LTD (Fig. 3o, r). These results suggest that fear memory was preferentially encoded in the cell ensembles manipulated with priming, which, however, was abolished with optical LTD delivered to the same synapses shortly after training.

### LTP alone shortly after FC changes neuronal ensembles encoding fear memory

**Fig. 4:**
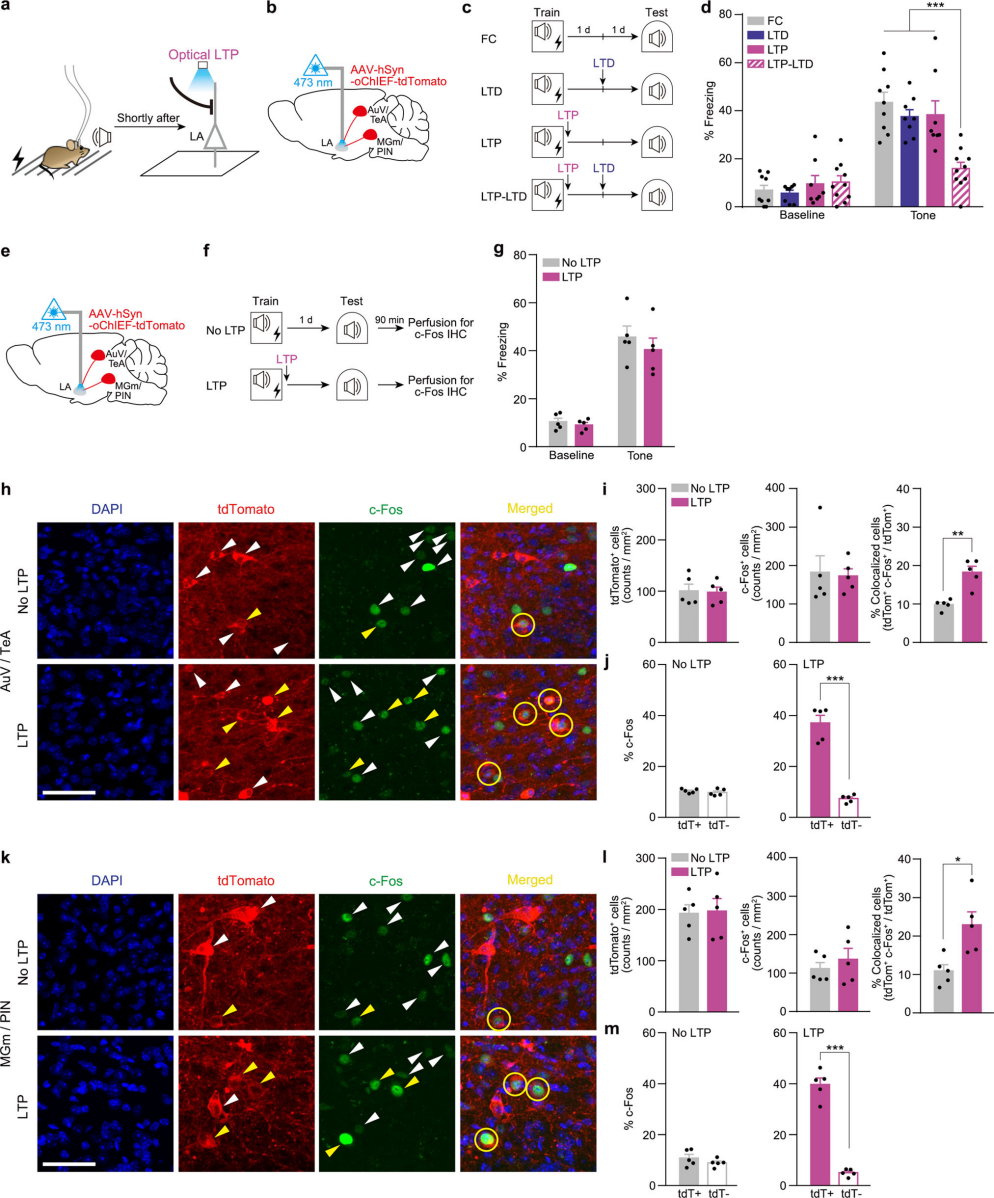
Fear memory is preferentially encoded in cell ensembles manipulated with optical LTP delivered shortly after training. **a** Schematic of behavior paradigm. Optical LTP was delivered shortly after FC. **b**, **e** AAV-hSyn-oChIEF-tdTomato was injected into the bilateral AuV/TeA and MGm/PIN with optic ferrule implantation above the LA. **c** Behavioral procedures. Optical LTP and LTD were delivered shortly and 24 h after FC, respectively. **d** Freezing levels to tone measured during test (FC, *n* = 9 mice; LTD and LTP, *n* = 8 mice per group; LTP-LTD, *n* = 10 mice; FC vs. LTP-LTD, LTP vs. LTP-LTD ***P < 0.0001; LTD vs. LTP-LTD ***P = 0.0001; Tukey’s post hoc test). **f** Behavioral procedures. Brains were taken 90 min after test for immunostaining. **g** Freezing levels to tone measured during Test (*n* = 5 mice per group). **h**, **k** Representative confocal microscopic images in the AuV/TeA (**h**) and MGm/PIN (**k**). Similar results were obtained from five independent mice per group, and the results were quantified in (**i**), (**j**), (**l**), (**m**). White arrows indicate tdTomato^+^ or c-Fos^+^ cells. Yellow arrows and circles indicate colocalized cells. Scale bar, 50 μm. **i**, **j**, **l**, **m** Quantitative analysis for distribution pattern of tdTomato^+^ and c-Fos^+^ cells in the AuV/TeA (**i**, **j**) and MGm/PIN (**l**, **m**). **i**, **l** Cell counts of tdTomato^+^ (left), c-Fos^+^ (middle), and tdTomato^+^/c-Fos^+^ overlap (right; **P = 0.001 (**i**), *P = 0.0133 (**l**); two-tailed unpaired *t* test). **j**, **m** c-Fos^+^ cells were more likely to be colocalized to tdTomato^+^ cells than neighboring tdTomato^-^ cells in LTP (right; ***P < 0.0001, two-tailed unpaired *t* test), but evenly distributed in No LTP control group (left). (*n* = 4 mice per group). Data are mean ± s.e.m. Two-way repeated-measures ANOVA (**d**, **g**). Two-tailed unpaired *t* test (**i**, **j**, **l**, **m**).

Finally, we asked whether even the optical LTP alone after normal FC training is sufficient to change engram (Fig. 4a). The AAV-oChIEF-tdTomato virus was injected into the bilateral AuV/TeA and MGm/PIN areas as before and the oChIEF-expressing axonal projections to the LA were optically targeted for LTP (Fig. 4b). To determine memory encoding at these synapses, we measured the effect of LTD delivered to these synapses 24 h post training on fear memory (Fig. 4c). Optical LTP was delivered shortly (~5 min) after FC training. Compared to a control group (FC), fear memory was normal in animals that received either optical LTP or LTD (Fig. 4d). In contrast, fear memory was severely impaired in animals that received optical LTD successively after LTP (Fig. 4d). To explore cells activated during memory recall in this condition, we again performed an imaging analysis as above. We compared the probability of activation of the labeled neurons (tdTomato^+^) in the AuV/TeA and MGm/PIN during memory retrieval between No LTP versus LTP conditions (Fig. 4e, f). As before, animals used for imaging analysis displayed no significant difference in freezing response to tone between groups during the test (Fig. 4g). From cell-counting analysis, we found that the overlap ratio between tdTomato^+^ and c-Fos^+^ cell population was significantly higher in the LTP group compared with No LTP control (Fig. 4h, i, k, l). Notably, there was no significant difference in the overall size of tdTomato^+^ and c-Fos^+^ cell populations between groups (Fig. 4i, l). The proportion of c-Fos^+^ cells was higher in tdTomato^+^ cells than in their noninfected neighbors in the LTP group, while the distribution was even in FC control (Fig. 4j, m). These results suggest that fear memory was preferentially encoded in the cell ensembles manipulated with optical LTP shortly after training.

## Discussion

It has been postulated that cell assemblies representing memory are formed by strengthening of synaptic connections between neurons through synaptic plasticity mechanisms such as long-term potentiation (LTP) ^12,13^. Our findings here support this idea. Prior studies show that strengthening is indeed observed at synaptic connections between interregional cell ensembles activated during learning and is crucial for memory retrieval^2,6,7,28^. Despite these findings, the role of synaptic plasticity for formation of engram during memory formation has been unclear. One possibility is that synaptic plasticity may be required for wiring neurons selected for memory encoding, but not involved in the neuronal selection itself. According to this view, it is assumed that synaptic plasticity manipulation after learning as we did in this study should not influence cell ensembles that participate in encoding memory, while it may affect consolidation or retrieval of memory. However, this was not what we observed in our study, so our data do not support this view. We instead propose that activity-dependent synaptic plasticity is involved in the selection of neurons for memory encoding. The constant size of c-Fos^+^ memory recall-activated cell population, regardless of plasticity manipulation, suggests that the selection process is competitive rather than cell autonomous. It has been shown previously that changes in CREB function influence the probability that individual LA neurons are recruited in encoding memory^29^. Interestingly, neurons expressing constitutively active CREB show facilitated long-term potentiation^30^.

It is unclear in this study how the artificial stimulations of inputs induced changes in neuronal ensembles. We hypothesize that any mechanism enabling persistent LTP formation at specific synapses is potentially involved in such ensemble changes. One possible scenario is that specific patterns of input activity may drive the selection of postsynaptic neurons, which in turn makes the specific input neurons selected through synapse-specific retrograde signaling mechanisms. According to this idea, the priming stimulation of inputs might lead to an increase in cellular excitability of the postsynaptic neurons, thereby making them preferentially recruited. As a potential mechanism, it has been previously shown in CA1 of rat hippocampal slices that a long-lasting increase in cellular excitability by activation of group 1 metabotropic glutamate receptors (mGluRs) can facilitate LTP induction^31^. Priming of trafficking of AMPA (α-amino-hydroxy-5-methyl-4-isoxazole propionic acid) receptors to the extrasynaptic membrane by mGluR-triggered trafficking of the mRNA for the AMPAR subunits GluA1 and GluA2 into dendrites is also a possible underlying priming mechanism^32–34^. If this is the case, it is assumed that the effects of the priming stimulus depend on group 1 mGluR signaling. According to the aforementioned scenario, postsynaptic engram cells could also be switched by the optic manipulations of presynaptic inputs. To examine the changes in LA ensembles by input activity, anterograde labeling of postsynaptic neurons in an input-specific manner would be necessary. For the retrograde information flow from postsynaptic to presynaptic neurons, transsynaptic retrograde signaling triggered from postsynaptic neurons is a strong candidate mechanism^35^. Previous studies using networks of cultured hippocampal neurons show a retrograde spread of LTD and LTP induced at glutamatergic synapses to the input synapses on the dendrite of the presynaptic neurons^36,37^. A feedback circuit from postsynaptic LA to auditory regions is another possible way that allows for retrograde information flow. A direct feedback circuit pathway from LA to the auditory cortex has been reported^38,39^. It is unclear whether there is a direct projection from the LA to the auditory thalamus, MGm/PIN. Given we previously found no reliable antidromic-firing of MGm or AuV by photostimulation of auditory axons in the LA^20^, back-propagating action potentials are unlikely to be involved in this process.

One limitation in our study is that the stimulation protocols we used do not accurately mimic the physiological activity patterns. For instance, in a natural condition, cortical inputs arrive in the LA a bit later than thalamic inputs^40^, but both inputs were simultaneously stimulated in our protocols. So, it is unclear whether activating both inputs at the same time is a necessary condition for driving a change in cell ensembles that participate in encoding memory. It is necessary to test in the future if manipulation of only one presynaptic input, either cortical or thalamic, can produce the same results. It is unknown whether only a portion of synapses among all activated by a tone is potentiated by fear conditioning, and priming mechanism is involved in such selection process. Interestingly, it has been previously shown in rats that acquisition of fear memory and in vitro induction of LTP in the LA requires mGluR5, one member of group 1 mGluRs whose activation can induce priming mechanisms as mentioned above^41,42^. To address if a natural tone activates a priming mechanism, a stimulation pattern that better mimics the physiological activity needs to be tested. Despite these limitations, bidirectional changes in cell ensembles by post learning LTD and LTP without affecting memory strongly support that memory encoding cell ensembles are formed by strengthening of synaptic connections between neurons.

It is striking in our study that memory allocation can be modified even after learning. One interpretation of our results is that neurons allocated into engram are not determined at the time of learning but rather there might be an ongoing synaptic activity-dependent competition process between eligible neurons for encoding by which engram is established over extended yet limited periods of time after an event. Through this process, the connections of selected neurons may stay strengthened whereas the ones of non-selected neurons end up being weakened, as in ocular dominance plasticity^43^. To achieve the competition, mechanisms mediating a complex cross-talk between synapses and neurons are likely involved. It has been reported in the CA1 region of the hippocampus that LTP induction in one set of afferents elicits a form of widespread heterosynaptic depression in an inactive, neighboring set of synapses that requires the spread of a signal between neurons^44^. It is possible that the optical LTP stimulation delivered shortly after FC might form LTP in the target postsynaptic LA neurons while inducing LTD in neighboring neurons through this type of widespread LTD mechanism. In this case, switching of engram cells is also expected in the LA. In addition, dynamic competitive interactions between synapses for plasticity factors have been reported^45,46^. These studies showed that when the availability of plasticity factors is limited, tagged synapses compete for them responsible for the persistent synaptic potentiation. Our findings suggest that neuronal ensembles can change by synaptic activities occurring after an event. We consider that such synaptic activities could be driven by another upcoming event or the internal activity resulting from the event itself. The encoding of two different events into overlapping cell populations has been suggested as a cell ensemble mechanism responsible for the memory linking^11,47^. The plasticity-dependent competition rule underlying memory formation therefore could provide a means for selective information storage and allow dynamic interactions between different events for memory encoding such as memory linking.

## Methods

### Mice

129S6 × C57Bl/6 hybrid mice (2–4-months-old, 22–35 g) were group-housed and kept on a 12-h light–dark cycle under constant temperature (21–23 °C) and humidity (40–60%). Food and water were supplied ad libitum throughout the experiments. All procedures were approved by the KAIST Institutional Animal Care and Use Committee.

### AAV production

AAV was packaged by cotransfection of DNA plasmids coding AAV 2/1, pAdΔF6 and viral vectors expressing opsins or reporters into HEK293T cells (ATCC, CRL-3216) and purification via an ultracentrifuge on an iodixanol gradient. Viral titers were measured by quantitative PCR (Qiagen, Rotor-gene Q and 204074) and ranged from 0.6 to 1.5 × 10^12^ Vg ml^−1^. For expression of ChR2, AAV-hSyn-ChR2-Venus vector was used. For expression of EGFP alone, AAV-hSyn-EGFP was used. For co-expression of ChR2 and eNpHR3.0-EYFP, we constructed AAV-hSyn-ChR2-2A-eNpHR3.0-EYFP. Separate ChR2 segment from the AAV-hSyn-ChR2-Venus and 2A-eNpHR3.0-EYFP segment from HSV-ChR2-2A-eNpHR3.0-EYFP were amplified by PCR and ligated into AAV vector to generate AAV-hSyn-ChR2-2A-eNpHR3.0-EYFP. For in vivo optical LTP induction, we used AAV-hSyn-oChIEF-tdTomato (Addgene plasmid # 50977). A complete list of the primer sets used in the quantitative PCR and subcloning are presented in Supplementary Table 1.

### Stereotaxic surgery

Mice were deeply anesthetized by intraperitoneal injection of sodium pentobarbital (83.3 mg kg^−1^) and fixed in a stereotaxic frame. Viruses were injected by using a glass micropipette attached to a 10-μl Hamilton microsyringe. A microsyringe pump and its controller were used to control the speed of injection. The glass pipette was slowly lowered to the target site and was left in place for an additional 10 min after injection to ensure diffusion. For bilateral AAV injection, a glass pipette containing AAV solution was targeted to both hemispheres (1 μl of AAV solution per each brain region) of auditory cortex, AuV and TeA (AP −2.7 mm, ML ± 4.45 mm, DV −3.2 mm), and auditory thalamus, MGm and PIN (AP −3.2 mm, ML ± 1.85 mm, DV −3.6 mm). For unilateral injection, AAV was injected into the left hemisphere. The AAV solutions were injected at a constant rate of 0.1 μl min^−1^. Optic ferrules (Doric Lenses) were implanted for optic fiber insertion by stereotaxic surgery 3 weeks after AAV injection. The ferrule tip was positioned 1 mm above the bilateral (AP −1.8 mm, ML ± 3.5 mm, DV −3.7 mm) or left side of LA (AP −1.8 mm, ML + 3.5 mm, DV −3.7 mm) and fixed with dental cement for chronic implantation. Ferrule-implanted mice were single-housed for a week before further experiments.

### In vivo-evoked field-potential (EFP) recording in anesthetized animal

EFPs were recorded in the LA using light stimulation of ChR2-expressing auditory inputs Three to 4 weeks after AAV-ChR2 or AAV-oChIEF injection, mice were anesthetized with isoflurane (4% for induction, 1% for maintenance) dissolved in mixed oxygen and nitrous oxide gas (3:7 ratio) and fixed in a stereotaxic frame. A commercially available optrode (Doric Lenses) with an electrode tip protruding 300 µm from the optic fiber tip was implanted into the left LA. EFP signals were amplified (×1000) and band-pass-filtered (10–1000 Hz) through a DAM80 differential amplifier (World Precision Instruments) and digitized at 10 kHz. Custom-written LabView 2011 code (National Instruments) was used for delivery of 470 nm LED light (0.4–1.2 mW at fiber tip, M470F1, Doric) or 473-nm laser light (0.3–15 mW at fiber tip, CL473-050-O, CrystaLaser) during recording and analysis of the processed data. The fEPSP slopes were calculated using a custom-written MATLAB code.

For each EFP recording session, ten EFPs were elicited by using 0.033 Hz light pulses for 5 min. Baseline EFPs were measured 24 h before the training procedure, and post EFPs were measured 5 min or 24 h after conditioning.

To confirm the optical LTD and LTP induction protocol in our condition^2^, we performed in vivo EFP recording using 473 nm laser light with 15 mW power at fiber tip (CL473-050-O, CrystaLaser). Mice were anesthetized with isoflurane as described above. The optrode was slowly lowered to the recording site. After stable baseline EFPs were established for 30 min (0.033 Hz light pulses), LTP was induced by using 5 trains of light (100 pulses per train, 100 Hz, 2 ms) with a 3-min intertrain interval. LTD was induced by using 900 light pulses (1 Hz, 2 ms). After delivery of light for LTP or LTD induction protocol, 60 or 120 EFPs were recorded (0.033 Hz), respectively.

### In vivo multiunit recording in anesthetized animal

#### Electroplating of the silicon probe

To massively acquire multiunit activities of the LA during light delivery, we used an optic fiber-coupled 16-channel silicon probe (A1x16-poly2-5mm-50s-177-OZ16LP, Neuronexus). The probe where impedance exceeded MΩ was consecutively electroplated with gold and PEDOT-TFB to lower impedance <200 kΩ^48^. To produce direct current (DC), a NanoZ (Multi Channel Systems) anode was connected to the probes and a cathode was connected to a stainless-steel bar. Both poles were flooded in 0.1 M NaClO_4_ containing 5 mM HAuCl_4_. Six brief pulses of DC (−0.1 μA, 2-s interval, 1-s pulse) were applied between the poles, and the resulting gold was plated onto the probe. For PEDOT-TFB coating, another solution (10 mM EDOT and 0.1 M tetrabutylammonium tetrafluoroborate in acetonitrile) was prepared and followed by ten pulses of DC (+0.03 μA, 5-s interval, 1-s pulse), resulting in deposition of PEDOT-TFB on the recording site. After electroplating, the impedance of the probes measured with NanoZ was 20-100 kΩ. A plated probe was used four to five times. Before each recording experiment, impedance was measured in PBS to confirm that it was in the proper range.

#### Multiunit recording and detection of neuronal firing

Mice injected with AAV-hSyn-ChR2-2A-eNpHR3.0-EYFP were anesthetized with isoflurane (4% for induction, 1% for maintenance) dissolved in mixed oxygen and nitrous oxide gas (3:7 ratio) and fixed in a stereotaxic frame. The impedance-matched probe was slowly lowered and placed in the LA recording site. The probe and ground/reference were connected to a PZ5 amplifier (Tucker-Davis Technologies) via a Zif-Clip adapter (ZCA32, Tucker-Davis Technologies). The amplified signal was delivered to and processed using a RZ5P processor (Tucker-Davis Technologies). The recording signal was sampled at 25 kHz and band-pass filtered at 300–5000 Hz. The neuronal firing was detected automatically by Synapse software (Tucker-Davis Technologies) when the spike amplitude was higher than 4 standard deviations from the median. The timing of neuronal firing was exported for further analysis. A customized open-source MATLAB code was used to draw raster plots. For drawing histograms, multiunit activities were binned at 0.5 Hz, with bins corresponding to different light activation conditions (no light, 473-nm light, 473-nm light, and 561-nm light). 473-nm laser light (CL473-050-O, CrystaLaser) was delivered to neurons in the AuV/TeA auditory cortex for their activation. To inhibit axonal inputs from those neurons in the LA, 561-nm laser light (CL561-050-O, CrystaLaser) was delivered to the LA via the optic fiber coupled to the probe. Both lasers were controlled by a RZ5P processor (Tucker-Davis Technologies) with a custom-made waveform using Synapse (Build 86-36093 M) software (Tucker-Davis Technologies). The 473-nm light (10 Hz, 20-ms pulse width) was delivered for 4 s, and continuous 561-nm light was presented simultaneously for the latter 2 s. The intensities of 473- and 561-nm light were 0.3 mW and 15 mW, respectively. The same light intensities were used for behavior experiments.

### Behavior

#### Auditory fear conditioning (FC)

All mice that were used for behavior experiments were habituated to the hands of the experimenter for 3 min on every 5 consecutive days. One day after the last day of habituation, auditory fear conditioning was performed in a metallic square chamber (178 × 178 × 305 mm, W × D × H, Coulbourn) with an electric grid floor. Seventy percent ethanol was used as a background odor. After 2 min of free exploration, tone (2.8 kHz, 85 dB) was presented for 30 s, which was co-terminated with a footshock (0.4 mA, 2 s). Mice remained in the training chamber for an additional 30 s after the footshock and then returned to their home cage. Tone test was performed in a context-shifted chamber with a non-glossy, half-rounded acrylic wall and flat floor serving as a distinct context. No background odor was used during any recall session. During the tone test, mice were placed in the test chamber for 2 min (baseline freezing) followed by 1-min of tone presentation (freezing to tone). For freezing measurement, the presence of optic fiber made it difficult to use automatic scoring of FreezeFrame software, so manual scoring was conducted by experienced experimenters in a blinded manner. In manual scoring, the time of immobility of the mouse without any movement except breathing was counted as freezing behavior. Using data recorded from control mice with no optic fiber equipped, we confirmed that the manual scoring gives a similar result with automatic scoring with FreezeFrame software. The same manual scoring method was used in a previously published paper^49^.

#### opto-FC training and test (Fig. 1g)

Mice injected with AAV-hSyn-ChR2-Venus were subjected to opto-FC training. After 2 min of free exploration on the training day, an opto-stimulation which consists of 10 trains of 473-nm light with 20-ms pulse width (each consisting of 20 pulses at 10 Hz, 0.3 mW) with a 1-s intertrain interval was delivered to the LA immediately before auditory fear conditioning. During the test, mice were placed in the chamber for 2 min (baseline freezing) followed by 1 min of 473-nm light stimulation (10 Hz, 20 ms). Five minutes afterward, these mice were tested with a 1-min tone presentation.

#### Opto-Trace-Shock training and test (Supplementary Fig. 1)

For Opto-Trace-Shock training, the same procedure as opto-FC was used except that tone was omitted. During the test, freezing responses to input photoactivation and tone were measured as in opto-FC.

#### Photoinhibition during the test (Fig. 1n–r and Supplementary Fig. 3)

Mice injected with AAV-hSyn-ChR2-2A-eNpHR3.0-EYFP were used for photoinhibition experiments. The mice were trained with opto-FC (Fig. 1o) or FC (Fig. 1q). During the test, mice were placed in the test chamber for 2 min (baseline freezing) followed by a 2-min tone presentation. For ON-OFF group, 56l-nm light was turned on during the first 1-min tone and subsequently turned off during the rest 1-min tone. For OFF-OFF control group, no light was illuminated during the entire 2-min tone session. For opto-FC (1-Hz) training (Supplementary Fig. 3b), the same procedure as opto-FC was used except that frequency of the opto-stimulation was changed to 1 Hz. For opto-5-min-FC training (Supplementary Fig. 3i), a 5-min interval was inserted between opto-stimulation and tone.

#### Anisomycin experiments (Fig. 1s–w and Supplementary Fig. 4)

Anisomycin (A9789, Sigma) was dissolved in 1 N HCl and diluted 1:1 in artificial cerebrospinal fluid (ACSF) to produce a final concentration of 125 µg µl^−1^. The pH was adjusted to 7.4 with 1 N NaOH. The solution was prepared freshly before each behavior experiment. Vehicle (ACSF) or anisomycin solution was infused into the bilateral LA (0.5 µl per side) immediately after the tone or photoactivation through an internal cannula (C313IS-5/Spc, Plastics One) projecting 1 mm from the guide cannula (3 mm C313GS-5/Spc, Plastics One) or the fluid injector of the fluid-optic cannula (FI OmFC-ZF1.25 100/170 4.8, Doric Lenses). Solutions were infused at a rate of 0.2 µl min^−1^. For the experiment in naive mice (Supplementary Fig. 4a), guide cannulas were bilaterally implanted above the LA (AP −1.8 mm, ML + 3.5 mm, DV −3.7 mm). Dummy cannulas (C313DCS-5/Spc, Plastics One) were used to prevent blood clot formation inside the guide cannulas. After recovery, mice underwent auditory fear conditioning. On the next day (day 2), vehicle or anisomycin solution was infused immediately following the tone presentation for 3 min. On the following day (day 3), the tone was presented during a memory recall test. For photoactivation experiments (Fig. 1t, v), Opto-fluid cannulas (OmFC_ZF1.25_200/245-0.37_4.5_FLT_3.8, Doric Lenses) were bilaterally implanted to deliver both fluid and light to the LA of bilaterally ChR2-injected mice. After recovery, mice underwent opto-FC or FC. On the next day (day 2), 473 nm light (10 Hz, 20 ms) was presented for 3 min, immediately after which vehicle or anisomycin solution was infused. On the following day (day 3), the tone was presented during the memory retrieval test. For no reactivation control experiment (Supplementary Fig. 4d), the same procedure as Fig. 1t was used, except that the photoactivation procedure was omitted.

#### Optical LTD (Figs. 2 and 3 and Supplementary Fig. 9)

For optical LTD induction to the auditory inputs in the LA after training (opto-FC or FC), LTD protocol (473 nm, 1 Hz, 900 pulses) was applied in a distinct context 1 d (Fig. 2), 30 min (Supplementary Fig. 9), or shortly (Fig. 3) after the training. To deliver optical LTD shortly after training, we transferred mice from a conditioning chamber to a distinct box immediately after training. Due to this transfer, there was an ~5 min interval to start the optical stimulation after conditioning. One day after the optical LTD, the freezing response to tone was measured. To test whether the optical LTD effect was specific to tone memory, we measured context freezing in the training context 1 day after optical LTD delivered 1 day after training (Fig. 2e). To test whether the LTD effect was permanent, we measured freezing response to tone again 14 days after training in the case of 1 d LTD (Fig. 2c, e, h). In No LTD control group, mice were placed in the same context used for LTD delivery in the LTD group but did not receive light. To further test the LTD effect in case that LTD was given shortly after the training, the second optical LTD was given 1 d after the first optical LTD (Fig. 3h).

#### Two-tone test (Supplementary Fig. 6)

As a distinguished tone from the conditioned tone (Tone1; 2800-Hz pure tone, 85 dB, 30 s), we used auditory pips (Tone2; 2 Hz pulses, 2-ms rise and fall, 7500 Hz, 85 dB, 30 s)^11^. Two groups of naïve mice were trained for auditory fear conditioning by using Tone1 or Tone2 (Tone1-US or Tone2-US, respectively) in the training chamber. One day after training, freezing response to Tone1 was measured in the test chamber with a shifted context. One day later, freezing response to Tone2 was measured in a distinct context different from Tone1 test context. Square acrylic wall and flat floor were used for the Tone2 test context.

#### Optical LTD and LTP (Fig. 2k and Supplementary Figs. 8b and 9f)

Tone test was performed twice before and after optical LTP protocol (100 Hz, 100 pulses in a single train, 5 trains, 3 min of an intertrain interval). One day (Fig. 2k), 30 min (Supplementary Fig. 8b) or shortly after (Supplementary Fig. 9f) the opto-FC, optical LTD protocol was delivered as before. One day later, freezing response to tone was measured (Test 1) and immediately followed by optical LTP protocol. One day after the LTP induction, freezing response was measured again (Test 2). In some cases, we measured freezing response to Tone2 (Test 3) 1 day after Test 2. To determine whether optical LTP to auditory inputs to the LA induces freezing response to tone (Supplementary Fig. 7), mice injected with AAV-hSyn-oChIEF-tdTomato were placed in a distinct context and received light (Supplementary Fig. 7b). For another control, the optical LTP in the distinct context was delivered immediately after the tone presented in the same context with tone test session (Supplementary Fig. 7d). In both cases, 1 day after the optical LTP, mice were tested with tone.

#### Optical LTP and LTD (Fig. 4d)

Optical LTP was delivered shortly after training in a distinct context. To deliver optical LTP, we transferred mice from a conditioning chamber to a distinct box immediately after training. Due to this transfer, there was an ~5 min interval to start the optical stimulation after conditioning. One day after the training, optical LTD was given in the same context where the optical LTP was given. Tone test was performed 24 h after the LTD induction.

### Histology

After behavior experiments, mice were sacrificed and brains were sliced into 40-mm coronal sections by Vibratome (Leica, VT1200S)^20^. Sections were observed under a fluorescence microscope for histological verification of virus expression and ferrule tip location (Supplementary Fig. 10). When AuV/TeA was mistargeted, expression was typically observed in the entorhinal cortex (ventral to the rhinal fissure). When MGm/PIN was mistargeted, medially biased expression was typically observed in the mesencephalic reticular formation or dentate gyrus. All off-target mice were excluded from data analysis. Specific AAV infection in auditory nuclei usually resulted in strong fluorescence signals in the LA. Of the mice with verified virus expression, only those showing no damage in the LA were included in the data analysis. Representative images were obtained using an LSM780 or LSM880 confocal microscope (Carl Zeiss). After the image acquisition, ZEN 3.1 software (Carl Zeiss) was used for modification of brightness or contrast.

### Immunohistochemistry and cell-counting analysis

To visualize c-Fos induction in auditory regions, mice were sacrificed 90 min after memory retrieval test (Figs. 3k and 4f). Brain slices (thickness of each slice, 40 μm; from AP −2.5 to −3.4 mm) separated by 120 μm were immunostained with rabbit anti-Fos antibody (1:5000, SC-52, Santa Cruz Biotechnology) followed by biotinylated goat anti-rabbit antibody (1:2000, 111-065-144, Jackson laboratory). The c-Fos signal was amplified by avidin–biotin peroxidase coupled with Fluorescein-TSA (NEL741001KT, PerkinElmer). In some cases, c-Fos immunostaining was performed with another rabbit anti-Fos antibody (1:2000, 226 003, Synaptic Systems). In these cases, Alexa Flour 488 anti-rabbit IgG (1:1000, A11008, Invitrogen) was used to visualize the c-Fos signal. Immunostained brain slices were loaded on a gelatin-coated slide glass and mounted with VECTASHIELD containing DAPI (h-1200, Vector). For each animal, three images adjacent to AAV virus injection sites in AuV/TeA and MGm/PIN were obtained using LSM880 confocal microscope (Carl Zeiss). Z-stack (step size: 2 μm) images were taken with ×20 objective (Carl Zeiss). tdTomato and c-Fos-positive cells in auditory regions were manually counted. Using Z-stack images, a clear tdTomato signal in cell membrane and cytosol were used to identify tdTomato-positive cells. tdTomato and c-Fos-positive cells in the auditory cortex and thalamus were counted from three neighboring brain slices which include AAV injection site. Based on mouse brain atlas^50^, the boundaries of the auditory cortex and thalamus were determined. Based on the boundaries, the area of the auditory cortex and thalamus in each slice was calculated by using ZEN 3.1 software (Carl Zeiss). To calculate the density of tdTomato or c-Fos positive cells, the total number of tdTomato or c-Fos positive cells in the cortex or thalamus of three brain slices from a single mouse was divided into the total area of the cortex or thalamus, then the values from each animal were averaged^51^. Overlap was calculated as an averaged ratio of the total number of tdTomato and c-Fos double-positive cells among the total number of tdTomato-positive cells of a single mouse. The proportion of c-Fos-positive cells among neighboring tdTomato-positive or -negative cells were calculated as the percentage of c-Fos and tdTomato double-positive cells/tdTomato-positive cells or c-Fos alone positive/tdTomato-negative DAPI-positive cells in randomly sampled windows where all two c-Fos and tdTomato signals were observed. To avoid any bias, the investigator was blinded to group allocation during the counting process, and any single-channel image was counted while hiding the other channels.

For the quantification of EGFP-expressing neurons (Supplementary Fig. 2), coronal brain sections (40-μm thickness, 120-μm interval) from five mice injected with AAV-hSyn-EGFP into the unilateral AuV/TeA and MGm/PIN were loaded on gelatin-coated slide glasses then mounted with VECTASHIELD containing DAPI (h-1200, Vector). Three brain sections closest from the virus injection site were taken in each mouse for cell-counting analysis. Z-stack (step size: 2 mm) images were taken using LSM880 confocal microscope (Carl Zeiss). We quantified the percentage of EGFP-expressing cells (EGFP-positive cells/DAPI-positive cells) in a randomly sampled window in each brain section where EGFP-positive soma was abundantly detected. For cell counting in the LA, three consecutive LA sections showing strong EGFP signals were selected in each mouse.

To determine protein expression of the IEGs in the LA (Supplementary Fig. 1f–j), brain slices including LA were immunostained with rabbit anti-Fos or anti-Arc antibodies (c-Fos: 1:2000, SC-52, Santa Cruz Biotechnology; Arc: 1:1000, 156,003, Synaptic Systems) followed by biotinylated goat anti-rabbit secondary antibody. IEGs’ signal was amplified by avidin–biotin peroxidase coupled with diaminobenzidine (D5905-50TAB, Sigma Aldrich) as a chromogen. Sections were loaded on a gelatin-coated slide glass and mounted with Cytoseal (8311-4, Thermo Scientific). IEG positive cells in the left LA were automatically counted from five coronal sections (from AP −1.4 to −2.3 mm) using computerized cell-counting software (NIS Elements software, Nikon). Cell density was calculated as the number of cells per mm^2^.

### Statistical analysis

The normality of every data set was confirmed via Shapiro–Wilk normality test (*α* = 0.05) using open-source MATLAB code. All other statistical analyses were performed using GraphPad Prism 8. Statistical significance was set at *P* < 0.05. All statistical results are presented in Supplementary Table 2.

## Supplementary information

Supplementary information

## Data Availability

All relevant raw data are available upon reasonable request from the corresponding authors. Source data are provided with this paper.

## Source data

Source Data

## Code availability

All custom codes used in this study are available from the corresponding author upon reasonable request.
