# Supplementary material for: Synaptic plasticity-dependent competition rule influences memory formation
Source: Nat Commun. 2021 Jun 24;12:3915. doi: 10.1038/s41467-021-24269-4 (PMC8225794; doi:10.1038/s41467-021-24269-4)
Supplement: Supplementary file 1 — Supplementary information [file 41467_2021_24269_MOESM1_ESM.pdf]

Synaptic plasticity-dependent competition rule influences memory formation

Supplementary Figures

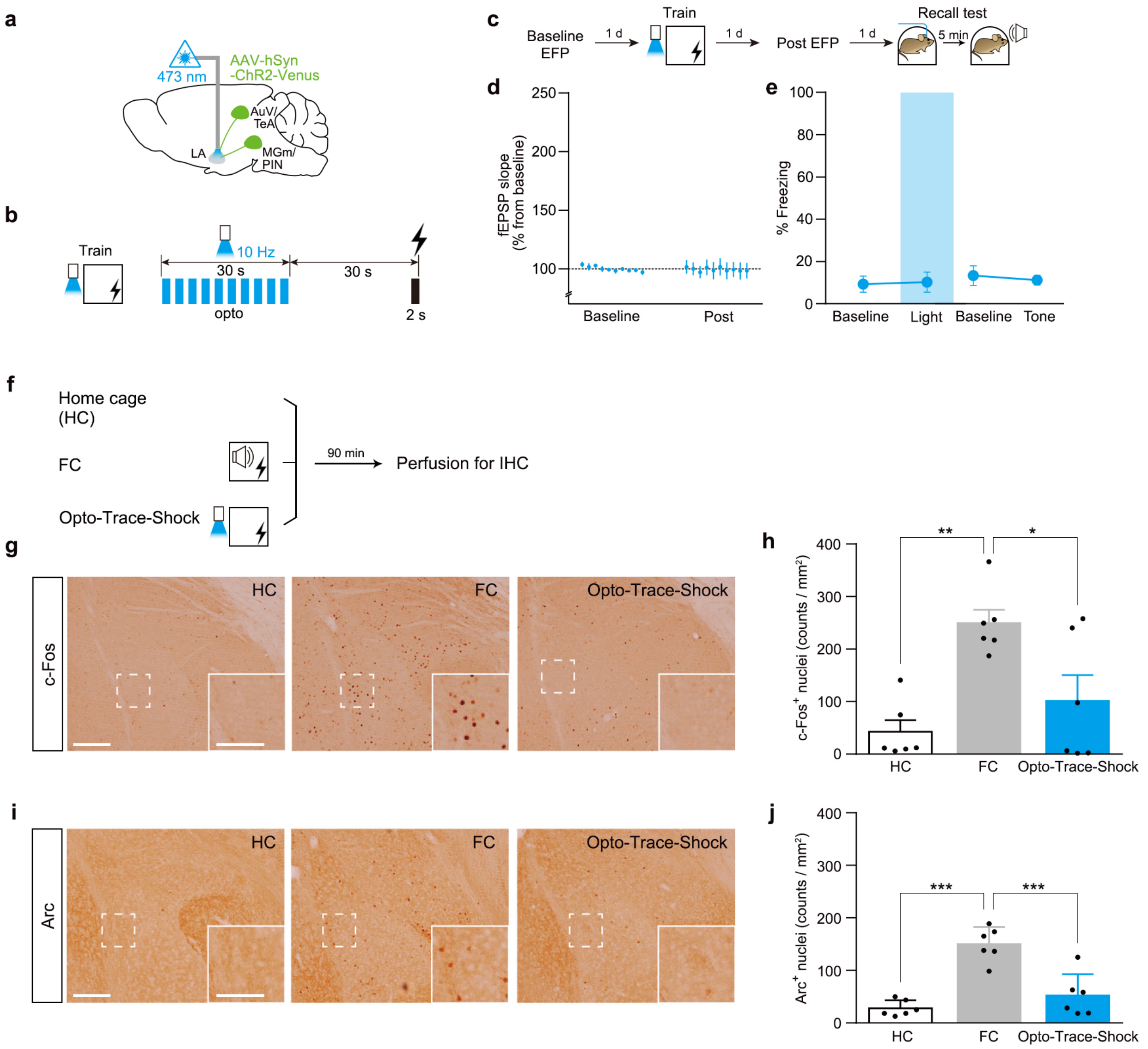

Supplementary Fig. 1 No LTP and long-term memory formation by pairing of a priming stimulus with a footshock.
a AAV-hSyn-ChR2-Venus was injected into the bilateral AuV/TeA and MGm/PIN with optic ferrule implantation above the LA. **b** Schematic of Opto-Trace-Shock paradigm. **c** Experimental procedure for EFP measurements and behavioral tests before and after Opto-Trace-Shock. **d** fEPSP slopes measured 1 d before (Baseline) and after (Post) training (n = 7 mice). **e** Freezing levels measured in response to the photo-stimulation (Blue shaded) and tone 1 d after post EFP recording (n = 7 mice). **f** Experimental procedures for IEG immunostaining. **g**, **i** Representative microscopic images of c-Fos (**g**) and Arc (**i**) immunostaining in the LA. Similar results were obtained from 6 independent mice per group, and the results were quantified in (**h**), (**j**). Scale bars, 200 µm (outer), 50 µm (inset). **h**, **j** Density of c-Fos$^+$ (**h**) or Arc$^+$ (**j**) nuclei in the LA (n = 6 mice per group; *P = 0.0169, **P = 0.0016, HC vs. FC ***P < 0.0001, FC vs. Opto-Trace-Shock ***P = 0.0002, Tukey's post-hoc test). Data are mean ± s.e.m. One-tailed two-way repeated measures (**d**, **e**) and one-way (**h**, **j**) ANOVA.

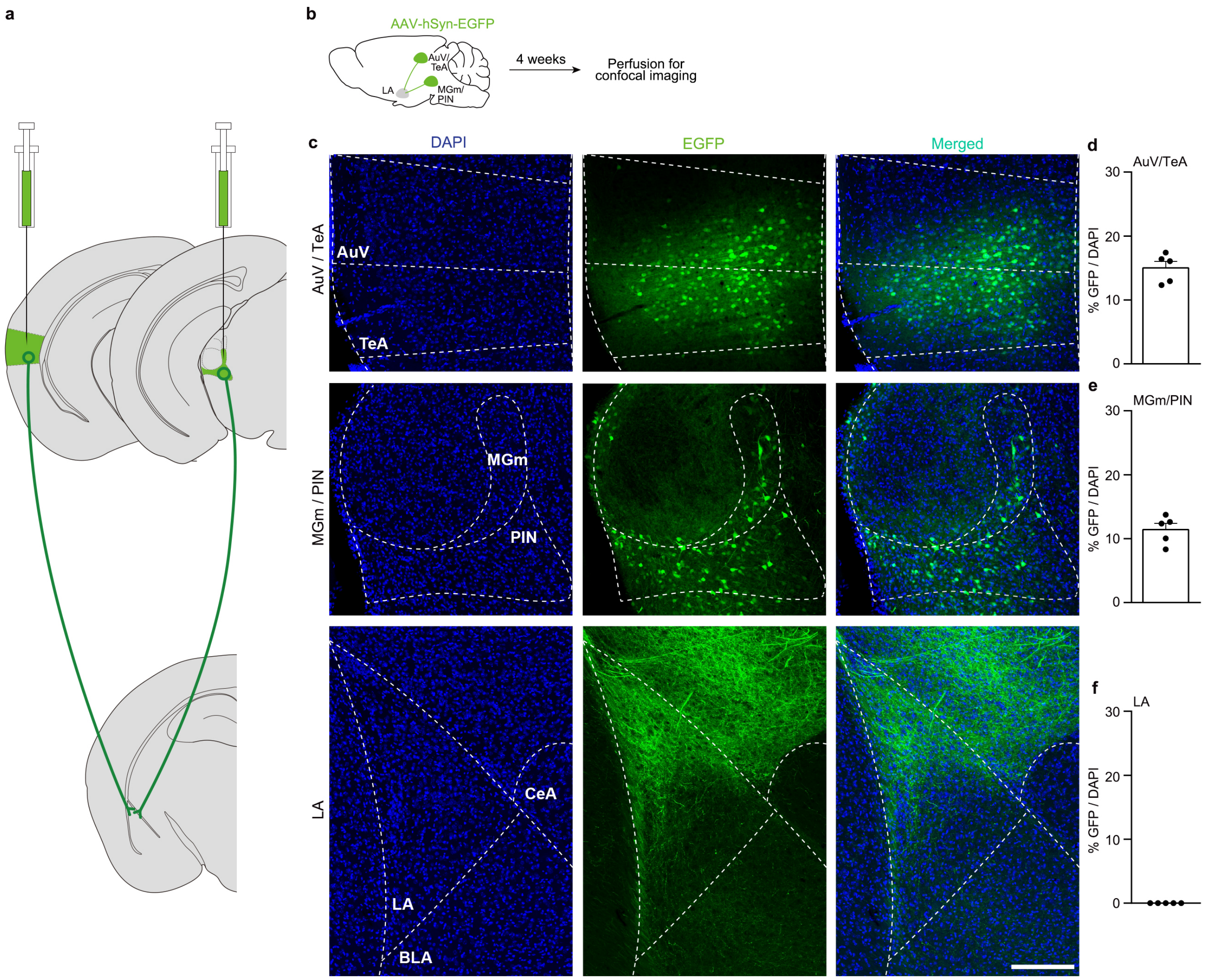

Supplementary Fig. 2 Expression pattern of EGFP in the auditory regions and LA in mice injected with AAV-hSyn-EGFP virus into the AuV/TeA and MGm/PIN.
a AAV-hSyn-EGFP virus (1.03 x 10^{12} Vg ml^{-1}) was injected into the unilateral AuV/TeA and MGm/PIN. **b** Four weeks after AAV-hSyn-EGFP injection, mice were perfused for confocal microscopic imaging analysis (n = 5 mice). **c** Representative confocal microscopic images. Scale bar, 200 μm. **d-f** Percentage of EGFP expressing cells ($EGFP^+/DAPI^+$) in the AuV/TeA (**d**), MGm/PIN (**e**), and LA (**f**) (n = 5 mice). Data are mean ± s.e.m.

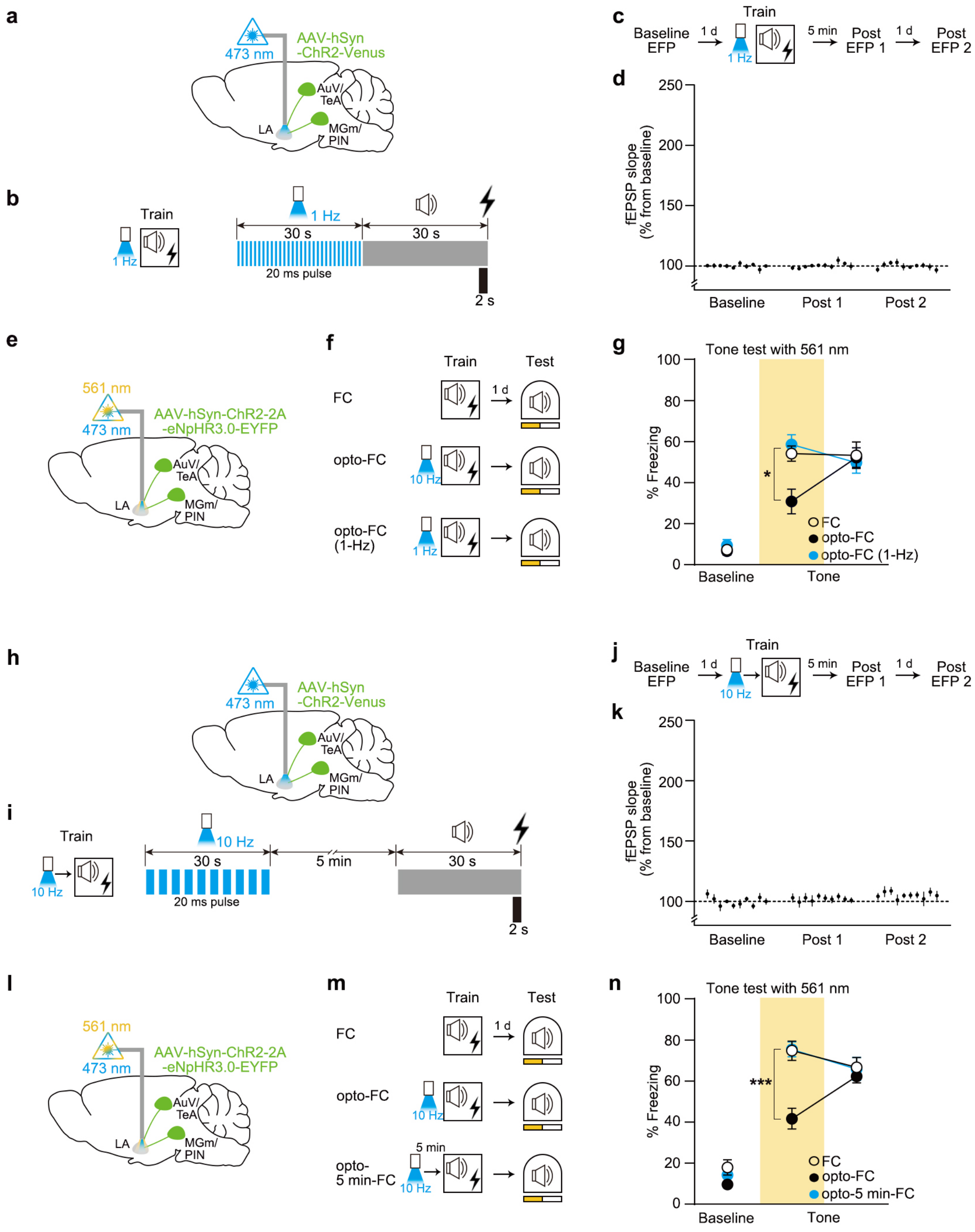

Supplementary Fig. 3 Silencing of the auditory inputs received non-priming opto-stimulation protocols does not disrupt fear memory recall.
a, **e**, **h**, **l** AAV-hSyn-ChR2-Venus (**a**, **h**) or AAV-hSyn-ChR2-2A-eNpHR3.0-EYFP (**e**, **l**) was injected into the bilateral AuV/TeA and MGm/PIN with optic ferrule implantation above the LA. **b** Schematic of opto-FC (1-Hz) protocol. The 1-Hz opto-stimulation preceded FC. **c** Experimental procedure for in vivo EFP recordings. **d** Average of in vivo fEPSP slope (normalized to baseline) 1 d before (Baseline), 5 min and 1 d after training (Post 1 and Post 2, respectively) (n = 6 mice). **f** Experimental procedures in each group. **g** Freezing levels measured during tone with (yellow shaded) or without 561 nm light (n = 6 mice per group). **i** Schematic of opto-5 min-FC protocol. The 10-Hz opto-stimulation preceded FC with 5 min interval. **j** Experimental procedure for in vivo EFP recordings. **k** Average of in vivo fEPSP slope (normalized to baseline) 1 d before (Baseline), 5 min and 1 d after training (Post 1 and Post 2, respectively) (n = 6 mice). **m** Experimental procedures in each group. **n** Freezing levels measured during tone with (yellow shaded) or without 561 nm light (n = 8 mice per group). Data are mean ± s.e.m. *P = 0.0149, ***P < 0.0001; two-way repeated measures ANOVA (**d**, **g**, **k**, **n**).

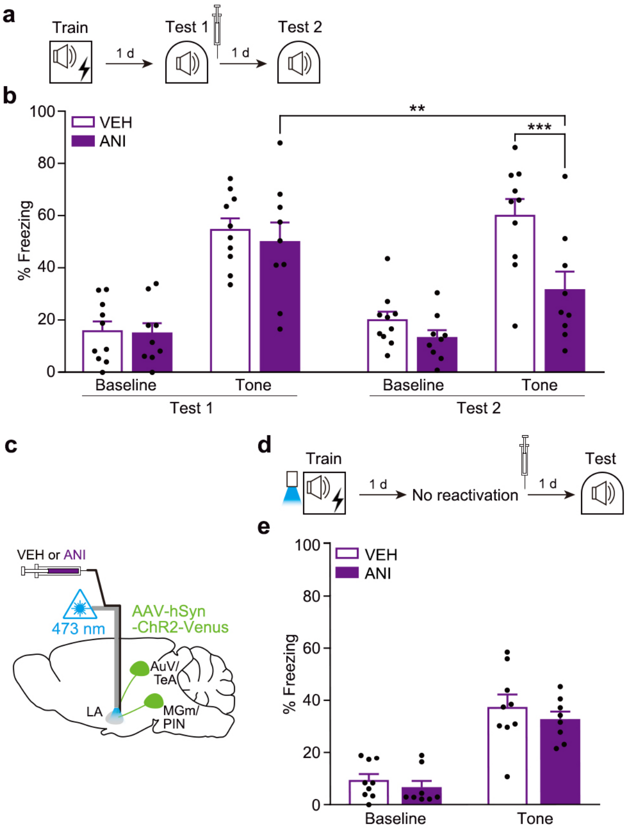

Supplementary Fig. 4 No amnesic effect of anisomycin without photo-stimulation of inputs. **a** Behavioral procedure. Mice were trained with FC. Anisomycin or vehicle was injected right after memory retrieval with tone. **b** Freezing levels to tone measured before (Test 1) and after (Test 2) drug administration (VEH, *n* = 10 mice; ANI, *n* = 9 mice). **c** Schematic of opto-stimulation and drug delivery in the bilateral LA of AAV-hSyn-ChR2-Venus injected mice. **d** Behavioral procedure. Mice were trained with opto-FC protocol. Anisomycin or vehicle was injected 1 day after training without photo-stimulation of the primed inputs. **e** Freezing levels to tone measured 1 day after drug administration (VEH, *n* = 9 mice; ANI, *n* = 8 mice). Data are mean ± s.e.m. $^{**}P = 0.0084$, $^{***}P = 0.0009$; two-way repeated measures ANOVA (**b**, **e**).

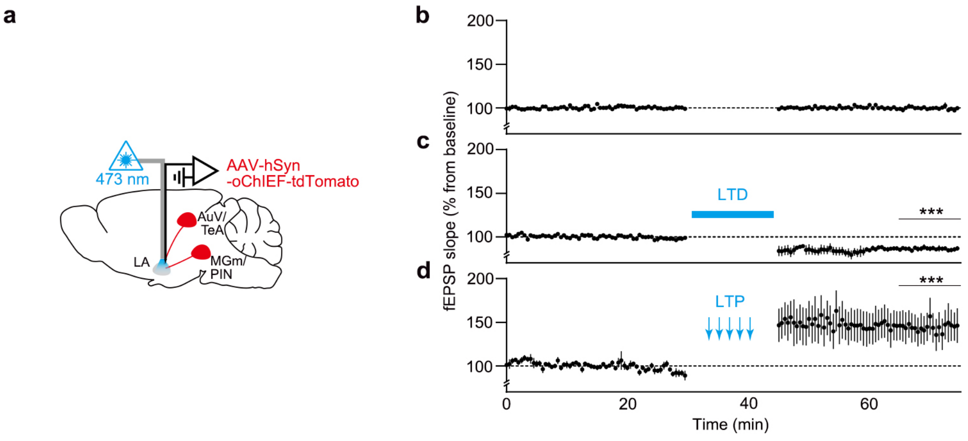

Supplementary Fig. 5 In vivo EFP responses to optical LTD and LTP protocols.
a AAV-hSyn-oChIEF-tdTomato was injected into the auditory regions and optrode was implanted above LA. Field responses were evoked by optical excitation of auditory inputs in the LA. **b-d** Average of in vivo fEPSP slope (normalized to baseline) before and after no stimulation (n = 4 mice) (**b**), LTD (n = 5 mice) (**c**), and LTP (n = 7 mice) (**d**) protocols. Data are presented as mean ± s.e.m. ***$P < 0.0001$; two-way repeated measures ANOVA (**b-d**).

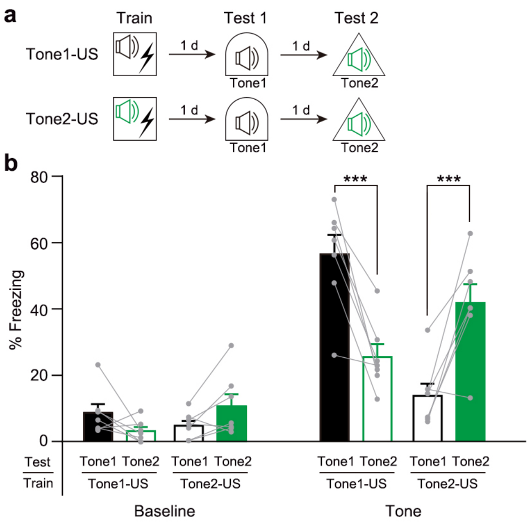

Supplementary Fig. 6 Auditory fear memory recall is specific to the tone used as a CS during training. **a** Behavioral procedures for Tone1-US and Tone2-US trainings followed by tests with Tone1 and Tone2. **b** Freezing levels in response to each tone during the tests ($n = 7$ mice per group). Data are presented as mean ± s.e.m. $^{***}P < 0.0001$; two-way repeated measures ANOVA.

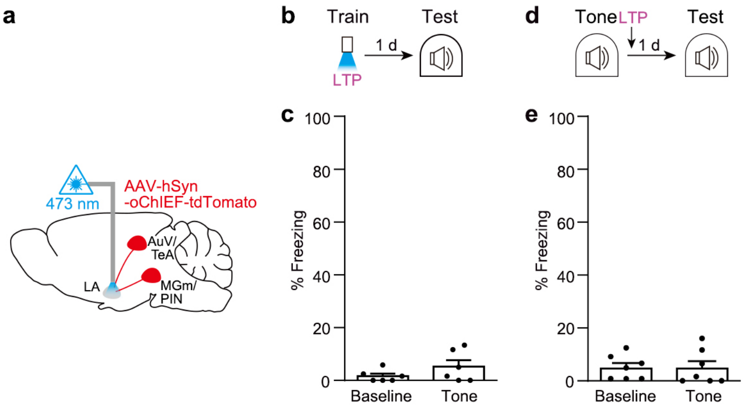

Supplementary Fig. 7 LTP induction alone at the auditory inputs does not elicit freezing to tone.
a AAV-hSyn-oChIEF-tdTomato was injected into the bilateral AuV/TeA and MGm/PIN with optic ferrule implantation above the LA. **b**, **d** Behavioral procedures. **c**, **e** Freezing levels measured before (Baseline) and during tone presentation (Tone) one day after optical LTP induction alone (**c**) or optical LTP following tone presentation (**e**). There was no significant freezing response to tone compared to baseline level (**c**, $n = 6$ mice; **e**, $n = 7$ mice). Data are mean ± s.e.m. Two-tailed paired t-test (**c**, **e**).

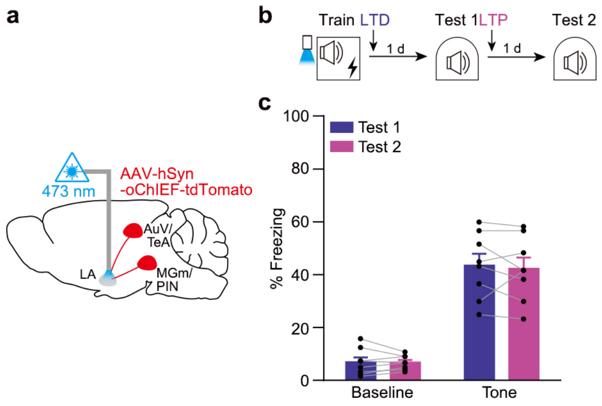

Supplementary Fig. 8 Optical LTP delivered 1 day after training does not affect fear memory in animals that are pre-exposed to optical LTD shortly after training.

a AAV-hSyn-oChIEF-tdTomato was injected into the bilateral AuV/TeA and MGm/PIN with optic ferrule implantation above the LA. **b** Behavioral procedure. **c** Freezing levels measured before (Baseline) and during (Tone) tone presentation in Test 1 and Test 2. There was no significant difference in freezing levels to tone between Test 1 and Test 2 (n = 8 mice). Data are mean ± s.e.m. Two-way repeated measures ANOVA.

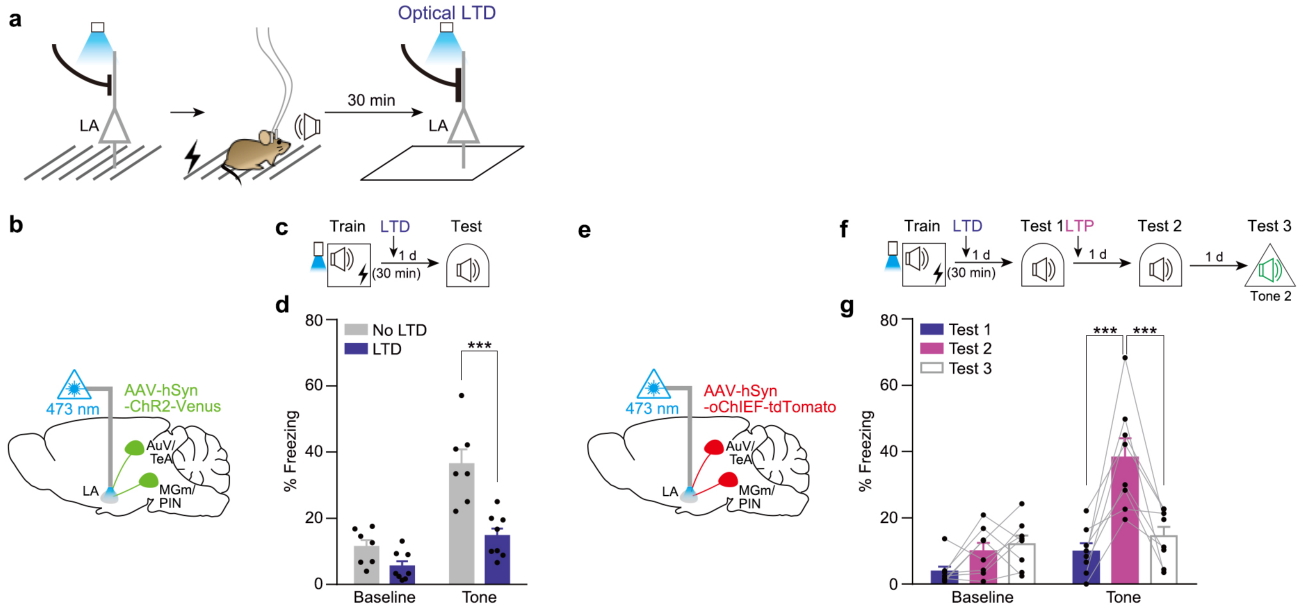

Supplementary Fig. 9 Optical LTD delivered to the primed inputs 30 min after training disrupts fear memory recall.

a Schematic of behavior paradigm. **b**, **e** AAV-hSyn-ChR2-Venus (**b**) or AAV-hSyn-oChIEF-tdTomato (**e**) was injected into the bilateral AuV/TeA and MGm/PIN with optic ferrule implantation above the LA. **c** Behavioral procedure. **d** Freezing levels to tone was significantly reduced in mice that received optical LTD (n = 8 mice) compared to control mice (n = 7 mice). **f** Behavioral procedure. Optical LTP was delivered right after Test 1 to mice that were exposed to optical LTD 30 min after training (n = 8 mice). **g** Freezing level to tone was measured during Tests. Tone 2 was presented as an unconditioned distinct tone in a distinct context. Data are presented as mean ± s.e.m. ***$P < 0.0001$; two-way repeated measures ANOVA (**d**, **g**).

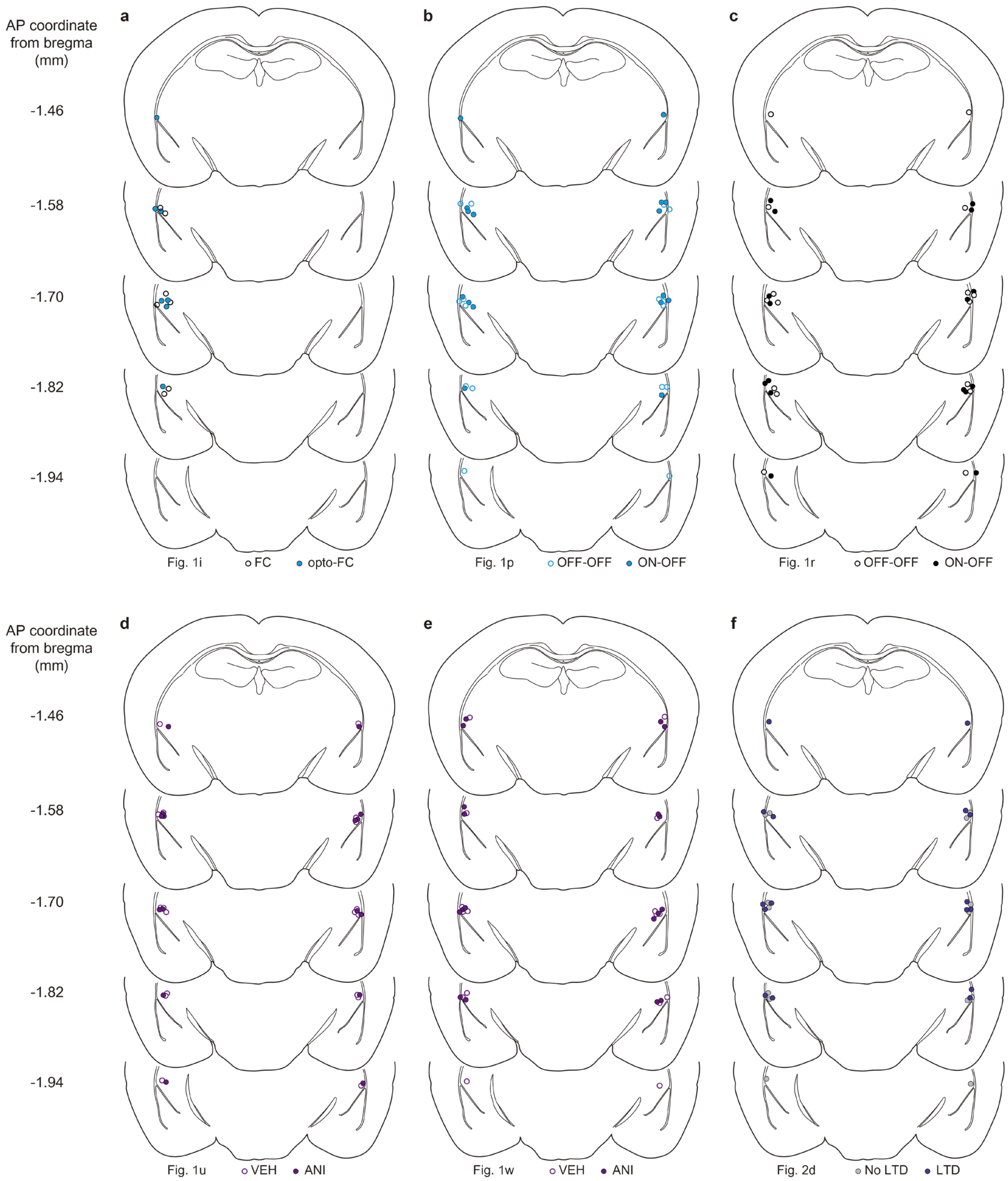
AP coordinate from bregma (mm)
-1.46
-1.58
-1.70
-1.82
-1.94
a
b
c
Fig. 1i
FC
opto-FC
Fig. 1p
OFF-OFF
ON-OFF
Fig. 1r
OFF-OFF
ON-OFF
AP coordinate from bregma (mm)
-1.46
-1.58
-1.70
-1.82
-1.94
d
e
f
Fig. 1u
VEH
ANI
Fig. 1w
VEH
ANI
Fig. 2d
No LTD
LTD

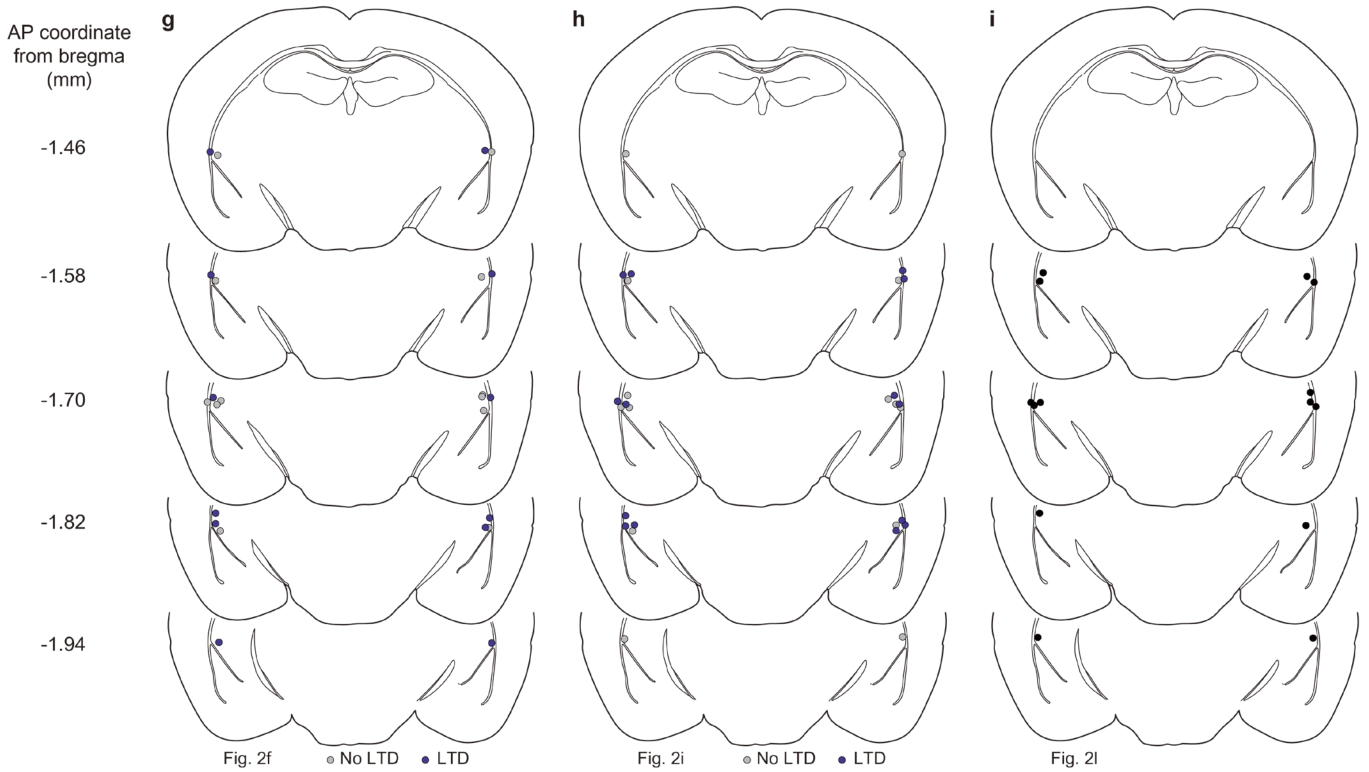
AP coordinate
from bregma
(mm)
-1.46
-1.58
-1.70
-1.82
-1.94
g
h
i
Fig. 2f
No LTD
LTD
Fig. 2i
No LTD
LTD
Fig. 2l

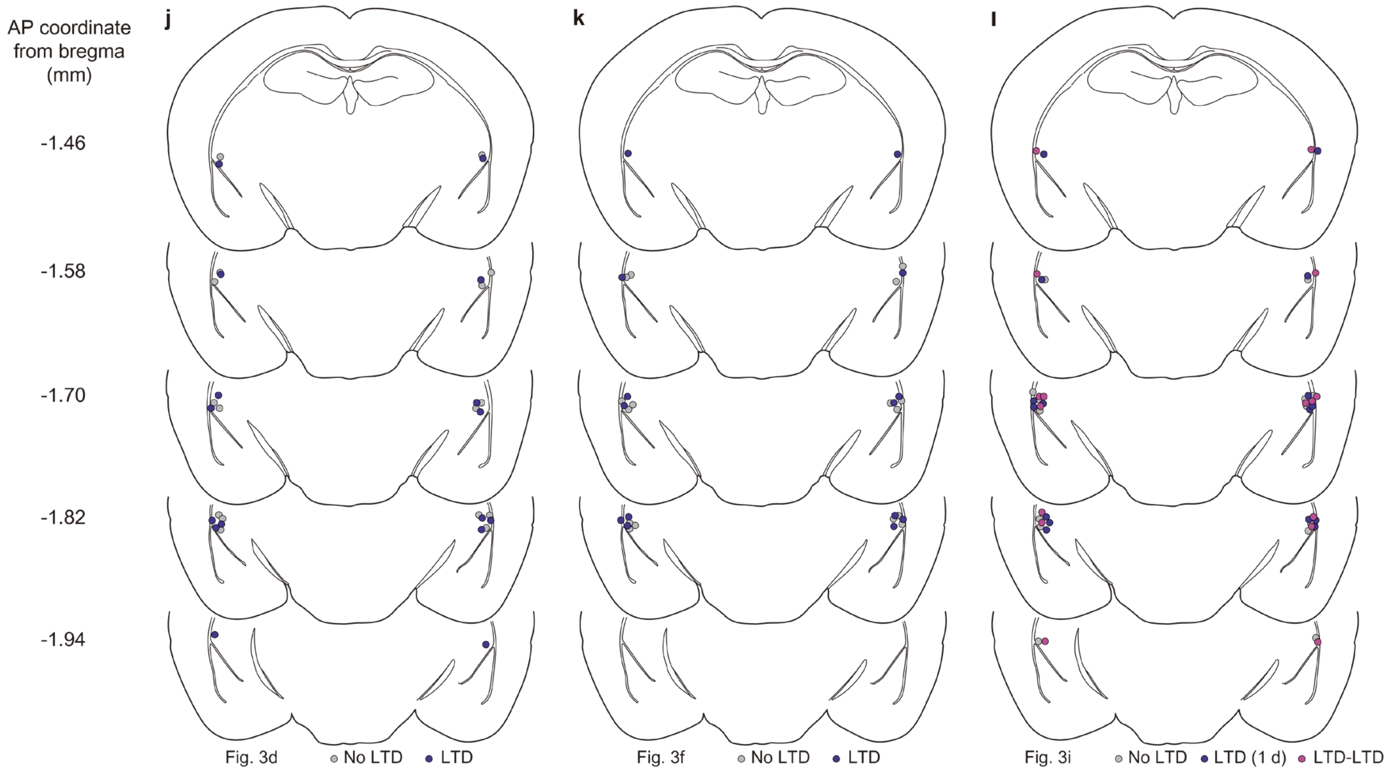
AP coordinate
from bregma
(mm)
-1.46
-1.58
-1.70
-1.82
-1.94
j
k
l
Fig. 3d
No LTD
LTD
Fig. 3f
No LTD
LTD
Fig. 3i
No LTD
LTD (1 d)
LTD-LTD

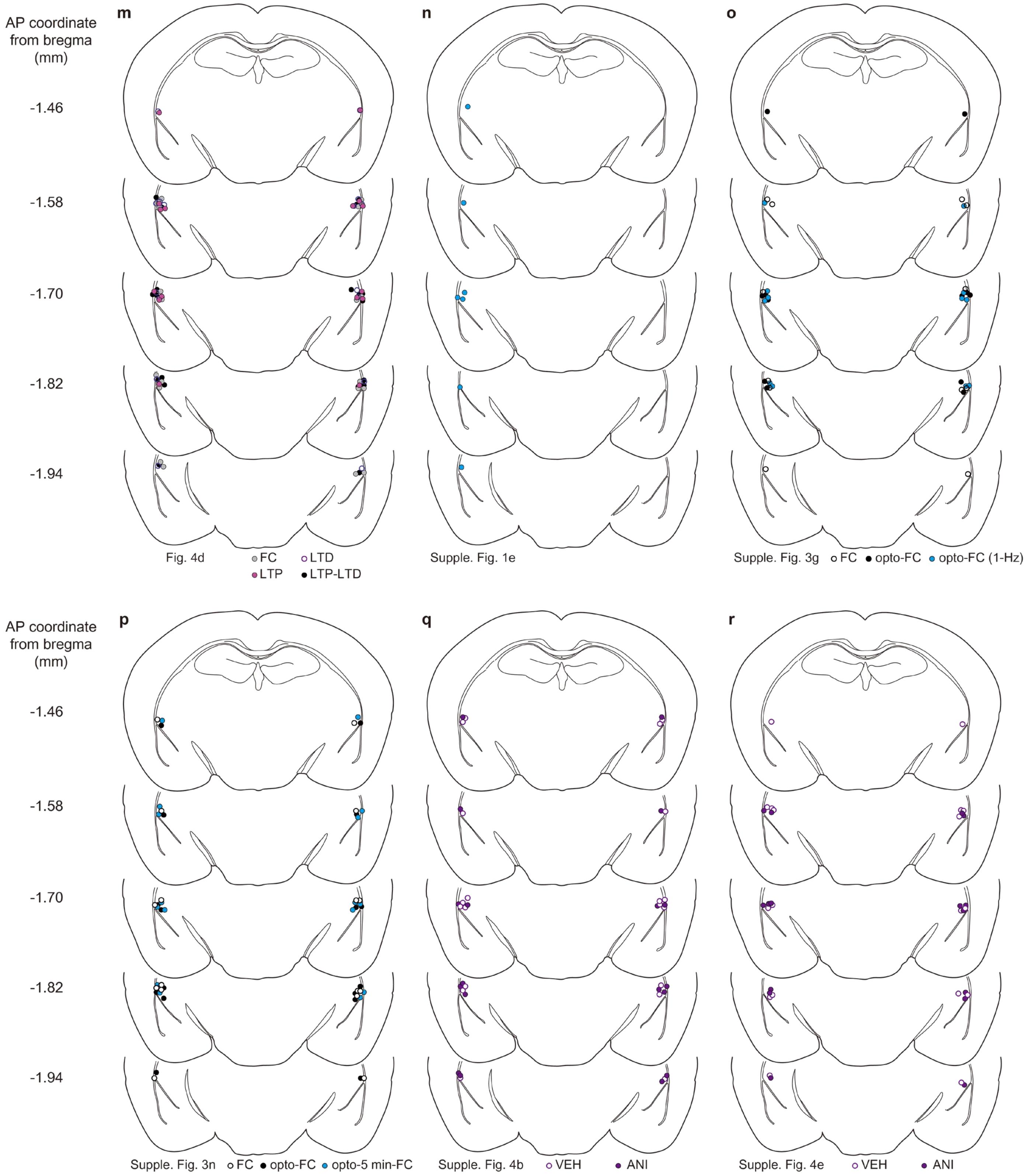

AP coordinate from bregma (mm)
-1.46
-1.58
-1.70
-1.82
-1.94
m
n
o
Fig. 4d
FC
LTD
LTP
LTP-LTD
Supple. Fig. 1e
Supple. Fig. 3g
FC
opto-FC
opto-FC (1-Hz)
AP coordinate from bregma (mm)
-1.46
-1.58
-1.70
-1.82
-1.94
p
q
r
Supple. Fig. 3n
FC
opto-FC
opto-5 min-FC
Supple. Fig. 4b
VEH
ANI
Supple. Fig. 4e
VEH
ANI

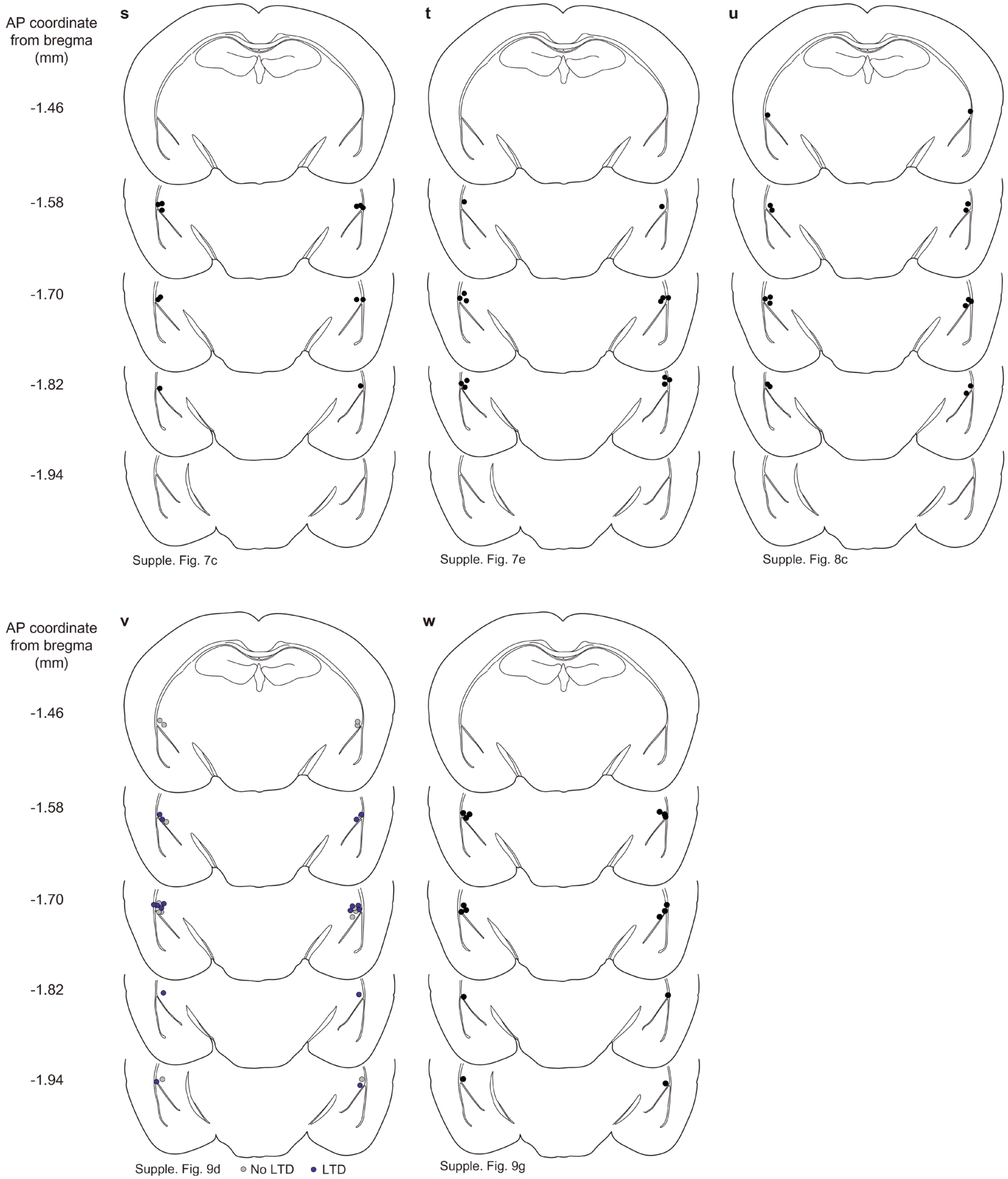

Supplementary Fig. 10 Histological verification of the locations of ferrule tips for behavior experiments with optogenetic manipulation.

Supplementary Table 1 Sequence and target of the primer sets used in the study.

Primer	Sequence (5' to 3')	Target	Direction
HS09	GATTCCAGGCATGCATGACC	Human growth hormone poly adenylation signal	Forward
HS10	CAGTGGTTCACGCCTGTAATC	Human growth hormone poly adenylation signal	Reverse
YR038	GCCGGTACCGAGGGCAGAGGAAGTCTTCT	2A-eNpHR3.0-EYFP	Forward
YR039	CGCAAGCTTTTACACCTCGTTCTCGTAGCA	2A-eNpHR3.0-EYFP	Reverse
YR041	AGCGCAGTCGAGAGCTAGC	ChR2	Forward
YR044	CGCGGTACCGACCGGTGGTTGCCGGTG	ChR2	Reverse

Supplementary Table 2 Detailed statistics in figures including sample size, statistical test used, *t*, *P*, and *F* values.

Figure	Group	Measurement variable	Statistical analysis	Effects of ANOVA	Statistical value	*P* value	Significance
Figure 1e	opto (n = 8)	% fEPSP slope	2-way RM ANOVA	Recording session (Baseline vs Post 1 vs Post 2)	$F_{2, 14}$ = 1.061	0.3722	
				Time point (1st to 10th in each session)	$F_{9, 63}$ = 0.7986	0.6188	
				Recording session × Time point	$F_{18, 126}$ = 1.416	0.1349	
	FC (n = 9)	% fEPSP slope	2-way RM ANOVA	Recording session (Baseline vs Post 1 vs Post 2)	$F_{2, 16}$ = 0.06123	0.9408	
				Time point (1st to 10th in each session)	$F_{9, 72}$ = 0.8135	0.6054	
				Recording session × Time point	$F_{18, 144}$ = 0.3501	0.9937	
	opto-FC (n = 8)	% fEPSP slope	2-way RM ANOVA	Recording session (Baseline vs Post 1 vs Post 2)	$F_{2, 14}$ = 11.98	0.0009	***
				Time point (1st to 10th in each session)	$F_{9, 63}$ = 1.345	0.2326	
				Recording session × Time point	$F_{18, 126}$ = 1.151	0.3126	
			Tukey's multiple comparison	Baseline vs Post 1		< 0.0001	***
				Baseline vs Post 2		< 0.0001	***
				Post 1 vs Post 2		0.1307	
Figure 1h	FC (n = 7)	% fEPSP slope	2-way RM ANOVA	Recording session (Baseline vs Post)	$F_{1, 6}$ = 0.1067	0.7550	
				Time point (1st to 10th in each session)	$F_{9, 54}$ = 0.7586	0.6543	
				Recording session × Time point	$F_{9, 54}$ = 0.3888	0.9354	
	opto-FC (n = 7)	% fEPSP slope	2-way RM ANOVA	Recording session (Baseline vs Post)	$F_{1, 6}$ = 8.580	0.0263	*
				Time point (1st to 10th in each session)	$F_{9, 54}$ = 1.151	0.3444	
				Recording session × Time point	$F_{9, 54}$ = 2.608	0.0141	*
			Sidak's multiple comparison	Baseline vs Post		< 0.0001	***
Figure 1i	FC (n = 7)	% Freezing	2-way RM ANOVA	Group (FC vs opto-FC)	$F_{1,12}$ = 0.4892	0.4976	
	opto-FC (n = 7)			Stimulus (Baseline vs Light or Baseline vs Tone)	$F_{3, 36}$ = 115.4	< 0.0001	***
				Group × Stimulus	$F_{3, 36}$ = 10.43	< 0.0001	***
			Sidak's multiple comparison	Light. FC vs opto-FC		0.0005	***
				Tone. FC vs opto-FC		0.1841	
			Tukey's multiple comparison	FC. Baseline vs Light		0.4730	
				FC. Baseline vs Tone		< 0.0001	***
				opto-FC. Baseline vs Light		< 0.0001	***
				opto-FC. Baseline vs Tone		< 0.0001	***
Figure 1p	OFF-OFF (n = 8)	% Freezing	2-way RM ANOVA	Group (OFF-OFF vs ON-OFF)	$F_{1,14}$ = 1.732	0.2093	
	ON-OFF (n = 8)			Stimulus (Baseline vs Tone+561 nm vs Tone)	$F_{2, 28}$ = 125.0	< 0.0001	***
				Group × Stimulus	$F_{2, 28}$ = 7.093	0.0032	**
			Sidak's multiple comparison	Tone+561 nm. OFF-OFF vs ON-OFF		0.0113	*
				Tone. OFF-OFF vs ON-OFF		0.9778	
			Tukey's multiple comparison	OFF-OFF. Baseline vs Tone+561nm		< 0.0001	***
				OFF-OFF. Baseline vs Tone		< 0.0001	***
				OFF-OFF. Tone+561nm vs Tone		0.2720	
				ON-OFF. Baseline vs Tone+561nm		< 0.0001	***
				ON-OFF. Baseline vs Tone		< 0.0001	***
				ON-OFF. Tone+561nm vs Tone		0.0323	*
Figure 1r	OFF-OFF (n = 8)	% Freezing	2-way RM ANOVA	Group (OFF-OFF vs ON-OFF)	$F_{1,14}$ = 0.09441	0.7632	
	ON-OFF (n = 8)			Stimulus (Baseline vs Tone+561 nm vs Tone)	$F_{2, 28}$ = 111.0	< 0.0001	***
				Group × Stimulus	$F_{2, 28}$ = 0.5197	0.6003	
			Tukey's multiple comparison	OFF-OFF. Baseline vs Tone+561nm		<0.0001	***
				OFF-OFF. Baseline vs Tone		<0.0001	***
				OFF-OFF. Tone+561nm vs Tone		0.0715	
				ON-OFF. Baseline vs Tone+561nm		<0.0001	***
				ON-OFF. Baseline vs Tone		<0.0001	***
				ON-OFF. Tone+561nm vs Tone		0.4797	
Figure 1u	VEH (n = 9)	% Freezing	2-way RM ANOVA	Group (VEH vs ANI)	$F_{1, 15}$ = 15.79	0.0012	**
	ANI (n = 8)			Stimulus (Baseline vs Tone)	$F_{1, 15}$ = 55.47	< 0.0001	***
				Group × Stimulus	$F_{1, 15}$ = 18.94	0.0006	***
			Sidak's multiple comparison	Tone. VEH vs ANI		< 0.0001	***
				VEH. Baseline vs Tone		< 0.0001	***
				ANI. Baseline vs Tone		0.0982	
Figure 1w	VEH (n = 8)	% Freezing	2-way RM ANOVA	Group (VEH vs ANI)	$F_{1, 15}$ = 0.6061	0.4484	
	ANI (n = 9)			Stimulus (Baseline vs Tone)	$F_{1, 15}$ = 176.5	< 0.0001	***
				Group × Stimulus	$F_{1, 15}$ = 0.8924	0.3598	
			Sidak's multiple comparison	VEH. Baseline vs Tone		< 0.0001	***
				ANI. Baseline vs Tone		< 0.0001	***
Figure 2d (Left)	No LTD (n = 7)	% Freezing	2-way RM ANOVA	Group (No LTD vs LTD)	$F_{1, 13}$ = 35.59	< 0.0001	***
	LTD (n = 8)			Stimulus (Baseline vs Tone)	$F_{1, 13}$ = 65.91	< 0.0001	***
				Group × Stimulus	$F_{1, 13}$ = 23.37	0.0003	***
			Sidak's multiple comparison	Tone. No LTD vs LTD		< 0.0001	***
				No LTD. Baseline vs Tone		< 0.0001	***
				LTD. Baseline vs Tone		0.0627	
Figure 2d (Right)	No LTD (n = 7)	% Freezing	2-way RM ANOVA	Group (No LTD vs LTD)	$F_{1, 13}$ = 40.44	< 0.0001	***
	LTD (n = 8)			Stimulus (Baseline vs Tone)	$F_{1, 13}$ = 104.4	< 0.0001	***
				Group × Stimulus	$F_{1, 13}$ = 45.59	< 0.0001	***
			Sidak's multiple comparison	Tone. No LTD vs LTD		< 0.0001	***
				No LTD. Baseline vs Tone		< 0.0001	***
				LTD. Baseline vs Tone		0.0491	*
Figure 2f	No LTD (n = 6)	% Freezing	Unpaired t test		t = 0.6881, df = 10	0.5070	
	LTD (n = 6)						
Figure 2i (Left)	No LTD (n = 7)	% Freezing	2-way RM ANOVA	Group (No LTD vs LTD)	$F_{1, 12}$ = 2.098	0.1731	
	LTD (n = 7)			Stimulus (Baseline vs Tone)	$F_{1, 12}$ = 226.2	< 0.0001	***
				Group × Stimulus	$F_{1, 12}$ = 1.969	0.1859	
			Sidak's multiple comparison	No LTD. Baseline vs Tone		< 0.0001	***
				LTD. Baseline vs Tone		< 0.0001	***
Figure 2i (Right)	No LTD (n = 7)	% Freezing	2-way RM ANOVA	Group (No LTD vs LTD)	$F_{1, 12}$ = 0.1082	0.7479	
	LTD (n = 7)			Stimulus (Baseline vs Tone)	$F_{1, 12}$ = 51.92	< 0.0001	***
				Group × Stimulus	$F_{1, 12}$ = 0.8188	0.3833	
			Sidak's multiple comparison	No LTD. Baseline vs Tone		0.0016	**
				LTD. Baseline vs Tone		0.0002	***

Figure	Group	Measurement variable	Statistical analysis	Effects of ANOVA	Statistical value	*P* value	Significance
Figure 2l	*n* = 7	% Freezing	2-way RM ANOVA	Test (Test 1 vs Test 2 vs Test 3)	$F_{2, 12}$ = 16.83	0.0003	***
				Stimulus (Baseline vs Tone)	$F_{1, 6}$ = 106.5	< 0.0001	***
				Test × Stimulus	$F_{2, 12}$ = 19.47	0.0002	***
			Sidak's multiple comparison	Tone. Test 1 vs Test 2		< 0.0001	***
				Tone. Test 1 vs Test 3		0.9998	
				Tone. Test 2 vs Test 3		< 0.0001	***
				Test 1. Baseline vs Tone		0.0693	
				Test 2. Baseline vs Tone		< 0.0001	***
				Test 3. Baseline vs Tone2		0.0260	*
Figure 3d	No LTD (*n* = 8)	% Freezing	2-way RM ANOVA	Group (No LTD vs LTD)	$F_{1, 14}$ = 0.7344	0.4059	
	LTD (*n* = 8)			Stimulus (Baseline vs Tone)	$F_{1, 14}$ = 116.1	< 0.0001	***
				Group × Stimulus	$F_{1, 14}$ = 0.003673	0.9525	
			Sidak's multiple comparison	No LTD. Baseline vs Tone		< 0.0001	***
				LTD. Baseline vs Tone		< 0.0001	***
Figure 3f	No LTD (*n* = 8)	% Freezing	2-way RM ANOVA	Group (No LTD vs LTD)	$F_{1, 13}$ = 1.681	0.2174	
	LTD (*n* = 7)			Stimulus (Baseline vs Tone)	$F_{1, 13}$ = 142.6	< 0.0001	***
				Group × Stimulus	$F_{1, 13}$ = 0.9170	0.3557	
			Sidak's multiple comparison	No LTD. Baseline vs Tone		< 0.0001	***
				LTD. Baseline vs Tone		< 0.0001	***
Figure 3i	No LTD (*n* = 7)	% Freezing	2-way RM ANOVA	Group (No LTD vs LTD vs LTD-LTD)	$F_{2, 20}$ = 11.67	0.0004	***
	LTD (1 d) (*n* = 8)			Stimulus (Baseline vs Tone)	$F_{1, 20}$ = 169.9	< 0.0001	***
	LTD-LTD (*n* = 8)			Group × Stimulus	$F_{2, 20}$ = 16.37	< 0.0001	***
			Sidak's multiple comparison	Tone. No LTD vs LTD (1 d)		< 0.0001	***
				Tone. No LTD vs LTD-LTD		0.1912	
				Tone. LTD (1 d) vs LTD-LTD		< 0.0001	***
				No LTD. Baseline vs Tone		< 0.0001	***
				LTD (1 d). Baseline vs Tone		0.0091	**
				LTD-LTD. Baseline vs Tone		< 0.0001	***
Figure 3l	FC (*n* = 4)	% Freezing	2-way RM ANOVA	Group (FC vs opto-FC vs opto-FC/LTD)	$F_{2, 9}$ = 0.8087	0.4754	
	opto-FC (*n* = 4)			Stimulus (Baseline vs Tone)	$F_{1, 9}$ = 106.6	< 0.0001	***
	opto-FC / LTD (n = 4)			Group × Stimulus	$F_{2, 9}$ = 0.4986	0.6232	
			Sidak's multiple comparison	FC. Baseline vs Tone		0.0008	***
				opto-FC. Baseline vs Tone		0.0013	**
				opto-FC/LTD. Baseline vs Tone		0.0002	***
Figure 3n (Left)	FC (*n* = 4)	$tdTomato^+$ cells	1-way ANOVA		$F_{2, 9}$ = 0.9011	0.4398	
	opto-FC (*n* = 4)						
	opto-FC / LTD (*n* = 4)						
Figure 3n (Middle)	FC (*n* = 4)	$c\text{-}Fos^+$ cells	1-way ANOVA		$F_{2, 9}$ = 0.4206	0.6689	
	opto-FC (*n* = 4)						
	opto-FC / LTD (*n* = 4)						
Figure 3n (Right)	FC (*n* = 4)	% colocalized cells	1-way ANOVA		$F_{2, 9}$ = 17.06	0.0009	***
	opto-FC (*n* = 4)		Tukey's multiple comparison	FC vs opto-FC		0.0010	**
	opto-FC / LTD (*n* = 4)			FC vs opto-FC / LTD		0.6056	
				opto-FC vs opto-FC / LTD		0.0038	**
Figure 3o (Left)	FC (*n* = 4)	% c-Fos	Unpaired t test		*t* = 0.3044, df = 6	0.7711	
Figure 3o (Middle)	opto-FC (*n* = 4)	% c-Fos	Unpaired t test		*t* = 13.60, df = 6	< 0.0001	***
Figure 3o (Right)	opto-FC / LTD (*n* = 4)	% c-Fos	Unpaired t test		*t* = 3.832, df = 6	0.0086	**
Figure 3q (Left)	FC (*n* = 4)	$tdTomato^+$ cells	1-way ANOVA		$F_{2, 9}$ = 0.7277	0.5094	
	opto-FC (*n* = 4)						
	opto-FC / LTD (*n* = 4)						
Figure 3q (Middle)	FC (*n* = 4)	$c\text{-}Fos^+$ cells	1-way ANOVA		$F_{2, 9}$ = 0.7677	0.4922	
	opto-FC (*n* = 4)						
	opto-FC / LTD (*n* = 4)						
Figure 3q (Right)	FC (*n* = 4)	% colocalized cells	1-way ANOVA		$F_{2, 9}$ = 17.87	0.0007	***
	opto-FC (*n* = 4)		Tukey's multiple comparison	FC vs opto-FC		0.0006	***
	opto-FC / LTD (*n* = 4)			FC vs opto-FC / LTD		0.0894	
				opto-FC vs opto-FC / LTD		0.0160	*
Figure 3r (Left)	FC (*n* = 4)	% c-Fos	Unpaired t test		*t* = 2.200, df = 6	0.0701	
Figure 3r (Middle)	opto-FC (*n* = 4)	% c-Fos	Unpaired t test		*t* = 14.60, df = 6	< 0.0001	***
Figure 3r (Right)	opto-FC / LTD (*n* = 4)	% c-Fos	Unpaired t test		*t* = 5.686, df = 6	0.0013	**
Figure 4d	FC (*n* = 9)	% Freezing	2-way RM ANOVA	Group (FC vs LTD vs LTP vs LTP-LTD)	$F_{3, 31}$ = 4.282	0.0208	*
	LTD (*n* = 8)			Stimulus (Baseline vs Tone)	$F_{1, 31}$ = 187.6	< 0.0001	***
	LTP (*n* = 8)			Group × Stimulus	$F_{3, 31}$ = 14.82	< 0.0001	***
	LTP-LTD (*n* = 10)		Tukey's multiple comparison	Tone. FC vs LTD		0.5977	
				Tone. FC vs LTP		0.7056	
				Tone. FC vs LTP-LTD		< 0.0001	***
				Tone. LTD vs LTP		0.9982	
				Tone. LTD vs LTP-LTD		0.0001	***
				Tone. LTP vs LTP-LTD		< 0.0001	***
			Sidak's multiple comparison	FC. Baseline vs Tone		< 0.0001	***
				LTD. Baseline vs Tone		< 0.0001	***
				LTP. Baseline vs Tone		< 0.0001	***
				LTP-LTD. Baseline vs Tone		0.3902	
Figure 4g	No LTP (*n* = 5)	% Freezing	2-way RM ANOVA	Group (No LTP vs LTP)	$F_{1, 8}$ = 0.8010	0.3969	
	LTP (*n* = 5)			Stimulus (Baseline vs Tone)	$F_{1, 8}$ = 111.2	< 0.0001	***
				Group × Stimulus	$F_{1, 8}$ = 0.3776	0.5560	
			Sidak's multiple comparison	No LTP. Baseline vs Tone		< 0.0001	***
				LTP. Baseline vs Tone		0.0002	***

Figure	Group	Measurement variable	Statistical analysis	Effects of ANOVA	Statistical value	*P* value	Significance
Figure 4i (Left)	No LTP (n = 5)	tdTomato$^+$ cells	Unpaired t test		t = 0.1793, df = 8	0.8622	
	LTP (n = 5)						
Figure 4i (Middle)	No LTP (n = 5)	c-Fos$^+$ cells	Unpaired t test		t = 0.1963, df = 8	0.8493	
	LTP (n = 5)						
Figure 4i (Right)	No LTP (n = 5)	% colocalized cells	Unpaired t test		t = 5.011, df = 8	0.0010	**
	LTP (n = 5)						
Figure 4j (Left)	No LTP (n = 5)	% c-Fos	Unpaired t test		t = 0.8247, df = 8	0.4334	
Figure 4j (Right)	LTP (n = 5)	% c-Fos	Unpaired t test		t = 9.783, df = 8	< 0.0001	***
Figure 4l (Left)	No LTP (n = 5)	tdTomato$^+$ cells	Unpaired t test		t = 0.1625, df = 8	0.8750	
	LTP (n = 5)						
Figure 4l (Middle)	No LTP (n = 5)	c-Fos$^+$ cells	Unpaired t test		t = 0.7364, df = 8	0.4825	
	LTP (n = 5)						
Figure 4l (Right)	No LTP (n = 5)	% colocalized cells	Unpaired t test		t = 3.163, df = 8	0.0133	*
	LTP (n = 5)						
Figure 4m (Left)	No LTP (n = 5)	% c-Fos	Unpaired t test		t = 1.181, df = 8	0.2714	
Figure 4m (Right)	LTP (n = 5)	% c-Fos	Unpaired t test		t = 13.11, df = 8	< 0.0001	***
Supple. Figure 1d	n = 7	% fEPSP slope	2-way RM ANOVA	Recording session (Baseline vs Post)	$F_{1, 6}$ = 0.07172	0.7978	
				Time point (1st to 10th in each session)	$F_{9, 54}$ = 0.6513	0.7482	
				Recording session × Time point	$F_{9, 54}$ = 0.9584	0.4840	
Supple. Figure 1e	n = 7	% Freezing	2-way RM ANOVA	Test (Test with the Light vs Test with the Tone)	$F_{1, 6}$ = 0.3946	0.5530	
				Stimulus (Baseline vs Light or Baseline vs Tone)	$F_{1, 6}$ = 0.09025	0.7740	
				Test × Stimulus	$F_{1, 6}$ = 0.4946	0.5082	
Supple. Figure 1h	HC (n = 6)	c-Fos$^+$ nuclei	1-way ANOVA		$F_{2, 15}$ = 10.01	0.0017	**
	FC (n =6)		Tukey's multiple comparison	HC vs FC		0.0016	**
	Opto-Trace-Shock (n = 6)			HC vs Opto-Trace-Shock		0.4878	
				FC vs Opto-Trace-Shock		0.0169	*
Supple. Figure 1j	HC (n = 6)	Arc$^+$ nuclei	1-way ANOVA		$F_{2, 15}$ = 25.50	< 0.0001	***
	FC (n =6)		Tukey's multiple comparison	HC vs FC		< 0.0001	***
	Opto-Trace-Shock (n = 6)			HC vs Opto-Trace-Shock		0.4031	
				FC vs Opto-Trace-Shock		0.0002	***
Supple. Figure 3d	n = 6	% fEPSP slope	2-way RM ANOVA	Recording session (Baseline vs Post 1 vs Post 2)	$F_{2, 10}$ = 0.05433	0.9474	
				Time point (1st to 10th in each session)	$F_{9, 45}$ = 0.9246	0.5132	
				Recording session × Time point	$F_{18, 90}$ = 1.170	0.3028	
Supple. Figure 3g	FC (n = 6)	% Freezing	2-way RM ANOVA	Group (FC vs opto-FC vs opto-FC (1-Hz))	$F_{2,15}$ = 2.155	0.1504	
	opto-FC (n = 6)			Stimulus (Baseline vs Tone+561 nm vs Tone)	$F_{2, 30}$ = 158.2	< 0.0001	***
	opto-FC (1-Hz) (n = 6)			Group × Stimulus	$F_{4, 30}$ = 6.635	0.0006	***
			Tukey's multiple comparison	Tone+561 nm. FC vs opto-FC		0.0149	*
				Tone+561 nm. FC vs opto-FC (1-Hz)		0.9984	
				Tone+561 nm. opto-FC vs opto-FC (1-Hz)		0.0019	**
				Tone. FC vs opto-FC		> 0.9999	
				Tone. FC vs opto-FC (1-Hz)		0.9997	
				Tone. opto-FC vs opto-FC (1-Hz)		> 0.9999	
				FC. Baseline vs Tone+561nm		< 0.0001	***
				FC. Baseline vs Tone		< 0.0001	***
				FC. Tone+561nm vs Tone		> 0.9999	
				opto-FC. Baseline vs Tone+561nm		0.0005	***
				opto-FC. Baseline vs Tone		< 0.0001	***
				opto-FC. Tone+561nm vs Tone		0.0026	**
				opto-FC (1-Hz). Baseline vs Tone+561nm		< 0.0001	***
				opto-FC (1-Hz). Baseline vs Tone		< 0.0001	***
				opto-FC (1-Hz). Tone+561nm vs Tone		0.6336	
Supple. Fig. 3k	n = 6	% fEPSP slope	2-way RM ANOVA	Recording session (Baseline vs Post 1 vs Post 2)	$F_{2, 10}$ = 2.212	0.1602	
				Time point (1st to 10th in each session)	$F_{9, 45}$ = 0.4736	0.8846	
				Recording session × Time point	$F_{18, 90}$ = 1.274	0.2240	
Supple. Fig. 3n	FC (n = 8)	% Freezing	2-way RM ANOVA	Group (FC vs opto-FC vs 5 min opto-FC)	$F_{2, 21}$ = 6.531	0.0062	**
	opto-FC (n = 8)			Stimulus (Baseline vs Tone+561 nm vs Tone)	$F_{2, 42}$ = 231.5	< 0.0001	***
	opto-5 min-FC (n = 8)			Group × Stimulus	$F_{4, 42}$ = 7.789	< 0.0001	***
			Tukey's multiple comparison	Tone+561 nm. FC vs opto-FC		< 0.0001	***
				Tone+561 nm. FC vs opto-5 min-FC		0.9998	
				Tone+561 nm. opto-FC vs opto-5 min-FC		< 0.0001	***
				Tone. FC vs opto-FC		0.9980	
				Tone. FC vs opto-5 min-FC		> 0.9999	
				Tone. opto-FC vs opto-5 min-FC		0.9996	
				FC. Baseline vs Tone+561nm		< 0.0001	***
				FC. Baseline vs Tone		< 0.0001	***
				FC. Tone+561nm vs Tone		0.9502	
				opto-FC. Baseline vs Tone+561nm		< 0.0001	***
				opto-FC. Baseline vs Tone		< 0.0001	***
				opto-FC. Tone+561nm vs Tone		0.0020	**
				opto-5 min-FC. Baseline vs Tone+561nm		< 0.0001	***
				opto-5 min-FC. Baseline vs Tone		< 0.0001	***
				opto-5 min-FC. Tone+561nm vs Tone		0.5138	
Supple. Fig. 4b	VEH (n = 10)	% Freezing	2-way RM ANOVA	Group (VEH vs ANI)	$F_{1, 17}$ = 3.323	0.0860	
	ANI (n = 9)			Stimulus (Baseline vs Tone in Test 1 and Test 2)	$F_{3, 51}$ = 51.20	< 0.0001	***
				Group × Stimulus	$F_{3, 51}$ = 5.336	0.0028	**
			Sidak's multiple comparison	Tone-Test 1. VEH vs ANI		0.9497	
				Tone-Test 2. VEH vs ANI		0.0009	***
			Tukey's multiple comparison	VEH. Test 1-Baseline vs Test 1-Tone		< 0.0001	***
				VEH. Test 1-Tone vs Test 2-Tone		0.7324	
				VEH. Test 2-Baseline vs Test 2-Tone		< 0.0001	***
				ANI. Test 1-Baseline vs Test 1-Tone		< 0.0001	***
				ANI. Test 1-Tone vs Test 2-Tone		0.0084	**
				ANI. Test 2-Baseline vs Test 2-Tone		0.0089	**

Figure	Group	Measurement variable	Statistical analysis	Effects of ANOVA	Statistical value	*P* value	Significance
Supple. Fig. 4e	VEH (*n* = 9)	% Freezing	2-way RM ANOVA	Group (VEH vs ANI)	$F_{1, 15}$ = 0.7523	0.3994	
	ANI (*n* = 8)			Stimulus (Baseline vs Tone)	$F_{1, 15}$ = 158.6	< 0.0001	***
				Group × Stimulus	$F_{1, 15}$ = 0.2017	0.6598	
			Sidak's multiple comparison	VEH. Baseline vs Tone		< 0.0001	***
				ANI. Baseline vs Tone		< 0.0001	***
Supple. Fig. 5b	No stimulation (*n* = 4)	% EFP slope	2-way RM ANOVA	Induction stage (Pre- vs Post-)	$F_{1, 3}$ = 0.02893	0.8758	
				Recording point (Last 20 points in each stage)	$F_{19, 57}$ = 0.5507	0.9245	
				Induction stage × Recording point	$F_{19, 57}$ = 0.6826	0.8201	
Supple. Fig. 5c	LTD (*n* = 5)	% EFP slope	2-way RM ANOVA	Induction stage (Pre- vs Post-)	$F_{1, 4}$ = 22.97	0.0087	**
				Recording point (Last 20 points in each stage)	$F_{19, 76}$ = 0.8196	0.6777	
				Induction stage × Recording point	$F_{19, 76}$ = 2.331	0.0050	**
			Sidak's multiple comparison	Pre-induction vs Post-induction		< 0.0001	***
Supple. Fig. 5d	LTP (*n* = 7)	% EFP slope	2-way RM ANOVA	Induction stage (Pre- vs Post-)	$F_{1, 6}$ = 7.589	0.0331	*
				Recording point (Last 20 points in each stage)	$F_{19, 114}$ = 0.9420	0.5338	
				Induction stage × Recording point	$F_{19, 114}$ = 1.225	0.2279	
			Sidak's multiple comparison	Pre-induction vs Post-induction		< 0.0001	***
Supple. Fig. 6b	Tone 1-US (*n* = 7)	% Freezing	2-way RM ANOVA	Group (Tone 1-US vs Tone 2-US)	$F_{1, 12}$ = 2.553	0.1361	
	Tone 2-US (*n* = 7)			Stimulus (Baseline vs Tone 1 vs Tone 2)	$F_{3, 36}$ = 44.52	<0.0001	***
				Group × Stimulus	$F_{3, 36}$ = 29.53	< 0.0001	***
			Sidak's multiple comparison	Tone1. Tone1-US vs Tone2-US		< 0.0001	***
				Tone2. Tone1-US vs Tone2-US		0.0187	*
			Tukey's multiple comparison	Tone1-US. Baseline vs Tone1		< 0.0001	***
				Tone1-US. Tone1 vs Tone2		< 0.0001	***
				Tone1-US. Baseline vs Tone2		0.0002	***
				Tone2-US. Baseline vs Tone1		0.2461	
				Tone2-US. Tone1 vs Tone2		< 0.0001	***
				Tone2-US. Baseline vs Tone2		< 0.0001	***
Supple. Fig. 7c	*n* = 6	% Freezing	Paired t test		*t* = 1.942, df = 5	0.1098	
Supple. Fig. 7e	*n* = 7	% Freezing	Paired t test		*t* = 0.01493, df = 6	0.9886	
Supple. Fig. 8c	*n* = 8	% Freezing	2-way RM ANOVA	Test (Test 1 vs Test 2)	$F_{1, 7}$ = 0.2275	0.6479	
				Stimulus (Baseline vs Tone)	$F_{1, 7}$ = 88.86	< 0.0001	***
				Test × Stimulus	$F_{1, 7}$ = 0.2702	0.6192	
			Sidak's multiple comparison	Test 1. Baseline vs Tone		< 0.0001	***
				Test 2. Baseline vs Tone		< 0.0001	***
Supple. Fig. 9d	No LTD (*n* = 7)	% Freezing	2-way RM ANOVA	Group (No LTD vs LTD)	$F_{1, 13}$ = 19.91	0.0006	***
	LTD (*n* = 8)			Stimulus (Baseline vs Tone)	$F_{1, 13}$ = 53.74	< 0.0001	***
				Group × Stimulus	$F_{1, 13}$ = 11.55	0.0048	**
			Sidak's multiple comparison	Tone. No LTD vs LTD		< 0.0001	***
				No LTD. Baseline vs Tone		< 0.0001	***
				LTD. Baseline vs Tone		0.0257	*
Supple. Fig. 9g	*n* = 8	% Freezing	2-way RM ANOVA	Test (Test 1 vs Test 2 vs Test 3)	$F_{2, 14}$ = 11.18	0.0013	**
				Stimulus (Baseline vs Tone)	$F_{1, 7}$ = 17.13	0.0044	**
				Test × Stimulus	$F_{2, 14}$ = 19.86	< 0.0001	***
			Sidak's multiple comparison	Tone. Test 1 vs Test 2		< 0.0001	***
				Tone. Test 1 vs Test 3		0.6568	
				Tone. Test 2 vs Test 3		< 0.0001	***
				Test 1. Baseline vs Tone		0.2185	
				Test 2. Baseline vs Tone		< 0.0001	***
				Test 3. Baseline vs Tone2		0.8460	
